# Supramolecular structures based on regioisomers of cinnamyl-α-cyclodextrins – new media for capillary separation techniques

**DOI:** 10.3762/bjoc.12.11

**Published:** 2016-01-19

**Authors:** Gabor Benkovics, Ondrej Hodek, Martina Havlikova, Zuzana Bosakova, Pavel Coufal, Milo Malanga, Eva Fenyvesi, Andras Darcsi, Szabolcs Beni, Jindrich Jindrich

**Affiliations:** 1 Department of Organic Chemistry, Faculty of Science, Charles University in Prague, Hlavova 8, 128 43, Prague 2, Czech Republic; 2 CycloLab, Cyclodextrin R&D Ltd, Budapest, H-1097 Illatos út 7, Hungary; 3 Department of Analytical Chemistry, Faculty of Science, Charles University in Prague, Hlavova 8, 128 43, Prague 2, Czech Republic; 4 Department of Pharmacognosy, Semmelweis University, Budapest, H-1085 Üllői út 26, Hungary

**Keywords:** capillary electrophoresis, cyclodextrin derivatives, mono-cinnamyl, regioisomers, supramolecular structures

## Abstract

This work focuses on the preparation and application of supramolecular structures based on mono-cinnamyl-α-cyclodextrins (Cin-α-CD). Pure regioisomers of Cin-α-CD having the cinnamyl moiety at the 2-*O-* or at the 3-*O*-position, respectively, were prepared, characterized and applied in capillary electrophoresis as additives to the background electrolyte. These new monomer units with a potential to self-organize into supramolecular structures were synthesized via a straightforward one-step synthetic procedure and purified using preparative reversed-phase chromatography allowing a large scale separation of the regioisomers. The ability of the monomers to self-assemble was proved by various methods including NMR spectroscopy and dynamic light scattering (DLS). The light scattering experiments showed that the monomer units have distinguishable ability to form supramolecular structures in different solvents and the size distribution of the aggregates in water can be easily modulated using different external stimuli, such as temperature or competitive guest molecules. The obtained results indicated that the two regioisomers of Cin-α-CD formed different supramolecular assemblies highlighting the fact that the position of the cinnamyl group plays an important role in the intermolecular complex formation.

## Introduction

Supramolecular polymers (SP) are aggregates of monomer units held together by non-covalent interactions, such as electrostatic interactions, coordination bonds, hydrogen bonds, hydrophobic interactions and host–guest interactions [1]. Their formation is spontaneous and reversible by self-assembly of the monomer units. Because of this special non-covalent intermolecular bonding, the formation and decomposition of the SP is at thermodynamic equilibrium, which means that the polymer growth or the destruction of the polymer chain can be adjusted by external stimuli. This reversibility makes SPs promising functional materials and gives them the potential to be easily processed, recycled or applied as self-healing materials. Recently much attention has been focused on the preparation and application of SPs and the most promising systems have already found their field of application ranging from cosmetics, printing, personal care to plastic industry. However their use as separation media in separation science remained an unexplored area [2].

The recent work focused on the preparation of supramolecular structures based on cyclodextrin (CD) [3] derivatives and on their application as chiral separation environment in capillary separation techniques. CDs are widely used in capillary separation techniques mainly as chiral selectors, but a common CD-based chiral selector does not form any superstructures and hence, may interact only with the analytes [4]. In comparison, the totally new type of molecular discriminators based on SPs generates superstructures (i.e., polymers) which also interact with analytes and moreover, exist and move as an entire aggregate in the separation environment. This character of the SPs brings an evident benefit in recognition, discrimination and consequently separation of analytes. Another advantage of the SP-based separation environment comes from the stimuli-responsivity of the system, which means that by adjusting different conditions of separation (temperature, ionic strength, pH, organic co-solvent concentration) it is possible to modulate the characteristics of the supramolecular assembly. The increasing or decreasing size of the SP will result in changed sieving effects, different selector–analyte interactions and in modulated separation selectivity.

CDs have been used as the host component for the construction of various interesting supramolecular structures such as pseudorotaxanes, rotaxanes, supramolecular dimers, oligomers and even polymers [5]. Modification of the parent CD molecule with an apolar substituent, which can form an inclusion complex with the CD’s cavity results in a conjugate with an ability to self-associate into supramolecular assemblies in polar solvents. The formation of these structures is based mainly on intermolecular host–guest interactions between the hydrophobic interior of the CD in one conjugate and between the apolar substituent of another conjugate. Earlier studies however pointed out that the size matching between the covalently attached guest part and the CD’s cavity is not the only requirement for the effective SP formation. In the case of 6-*O*-monobenzoyl-β-CD, for example, the direct attachment of the phenyl moiety to the CD rim did not result in intermolecular complex formation [6]. On the other hand too long and flexible spacers between the host and the guest moiety favored the self-inclusion process of the guest part to the parent CD’s cavity [6–7]. These findings showed that a compromise in the flexibility and in the length of the spacer has to be found for efficient formation of intermolecular complexes. Inspired by these works our approach towards SPs was based on the preparation of monocinnamyl-α-CDs, where the rigid double bond in the cinnamyl (Cin) moiety should prevent the self-inclusion of the phenyl ring to the CD cavity and prefer the formation of intermolecular complexes in polar solvents.

The conjugation of the cinnamoyl (Cio) moiety with α- and β-CD through amide and ester linkages was already reported by Harada et al. [8–9]. These monosubstituted derivatives formed different types of supramolecular assemblies (dimers, cyclic oligomers, linear polymers) in water depending on the position of the cinnamoyl moiety on the CD rim. It was also experimentally proven that the cavity of the CD shows distinguishable inclusion affinities towards different substrates, even to homologues and isomers. A self-sorting complex formation ability was described for the two regioisomers of monocinnamoyl-α-CD (Cio-α-CD) [9]. While the 2-Cio-α-CD isomer formed an insoluble double-threaded supramolecular dimer in water, the 3-Cio-α-CD formed a soluble supramolecular oligomer in the same solvent. The mixture of both regioisomers led to the formation of a self-sorting oligomeric system, where only the heterosupramolecular interactions between the two isomers were present but the homosupramolecular interactions between the same species were missing. This work clearly demonstrates that the intermolecular complex formation is a regioselective process where the CD cavity can differentiate between the positional isomers which will result in different aggregation behavior of the regioisomers.

Based on this knowledge our aim was to prepare pure regioisomers of cinnamyl-appended α-CD, to test and compare the ability of different regioisomers to form supramolecular structures and to investigate their application as additives to the background electrolyte in capillary separation techniques. Because this application requires chemically stable conjugates, we decided to prepare conjugates where the guest molecule is bound to the CD by a stable ether bond instead of the labile ester bond. This led us to the family of Cin-α-CD. The complete set of peracetylated regioisomers of Cin-α-CD was previously prepared and characterized by our research group, but our attempts to separate the single 2-*O*- and 3-*O*-regioisomers without further modification were unsuccessful [10]. In order to isolate the single regioisomers using this published method, exhaustive per-*O*-acetylation of the mixture of regioisomers, chromatographic separation of the per-*O*-acetylated derivatives and the de-*O*-acetylation of the separated per-*O*-acetylated regioisomers would be necessary. This three-step reaction procedure makes the large scale preparation of the single regioisomers expensive and time-consuming. For α-CD we were not able to achieve the high regiospecificity of the monocinnamylation which we described for β-CD earlier [11]. In the case of β-CD the only monosubstituted isomer obtained was 3-*O*-Cin-β-CD in 30% isolated yield. This result was explained by a highly regioselective complexation of the alkylation reagent into β-CD, which obviously does not take place with α-CD and only mixtures of regioisomers are formed.

Herein, we report on a straightforward one-step synthetic methodology using preparative reversed-phase chromatography with water–methanol gradient elution for the separation of the regioisomers of Cin-α-CD.

## Results and Discussion

The two regioisomers of Cin-α-CD were synthesized via direct alkylation of α-CD (**1**) in DMSO, using NaH as a base for the deprotonation of the secondary OH groups of the α-CD and cinnamyl bromide as an alkylating agent. The reaction resulted in a multicomponent mixture of unreacted α-CD, monosubstituted α-CD and disubstituted α-CD. From this mixture of compounds the pure 2-*O*-Cin-α-CD (**2**) and 3-*O*-Cin-α-CD (**3**) were isolated in one run by preparative reversed-phase chromatography using a water–methanol step gradient elution. This modified procedure allowed us to prepare the two regioisomers of Cin-α-CD in gram-scale which was required for the detailed characterization of their aggregation ability and for their application in capillary electrophoresis (CE). The monosubstituted products were characterized by ESI mass spectrometry, ^1^H NMR and ^13^C NMR spectroscopy.

**Figure 1:**
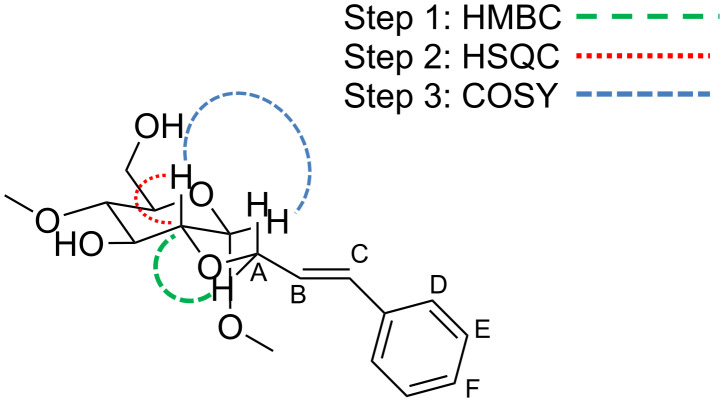
Example of elucidation of 2D NMR spectra of 2-*O*-Cin-α-CD.

The two regioisomers could be unambiguously distinguished and characterized by cross-linking the data of ^1^H NMR, ^13^C NMR, COSY, TOCSY, DEPT-ed-HSQC and HMBC. As an example, the elucidation of the regioisomer **2** is discussed here. The allylic proton signals can be detected in a separated region of the ^1^H proton spectrum around 4.47 ppm (see proton A in Figure SI 1, Supporting Information File 1). These frequencies can be also easily identified in the DEPT-ed-HSQC spectrum of the compound (see A in Figure SI 3, Supporting Information File 1). The DEPT-ed-HSQC spectrum clearly shows that the compound is unsubstituted at the C6 position, since the characteristic peaks of the two magnetically non-equivalent proton (C6-Ha and C6-Hb), typical for C6 substitution [12] are not detectable in the spectrum. As a consequence the compound is C2- or C3-monosubstituted (the monosubstitution has also been proven by MS). Analyzing the HMBC spectrum (Figure SI 4, Supporting Information File 1), crosspeaks between the allylic protons (4.47 ppm) and a CD-related carbon at 81.38 ppm can be detected (see the black circle in Figure SI 4, Supporting Information File 1). This carbon resonance (81.38 ppm) can either belong to C2’ or C3’ of the glucose unit bearing the cinnamyl substituent (otherwise the crosspeak would not be present in the HMBC spectrum). The frequency of this carbon (and of the correlated proton at 3.57 ppm) can be also detected in the DEPT-ed-HSQC spectrum (see C2’ in Figure SI 3, Supporting Information File 1). Finally, the COSY spectrum clarifies that the carbon at 81.38 ppm corresponds to C2’ since the crosspeak with the anomeric resonance can be detected (see the black circles in Figure SI 5, Supporting Information File 1). This set of data proves unambiguously that the compound is the 2-*O*-Cin-α-CD. A schematic representation of the spectral evidences used for the identification of the cinnamyl position is shown in Figure 1.

### Supramolecular structures of 2-*O*-Cin-α-CD and 3-*O*-Cin-α-CD characterized by 2D rotating frame Overhauser effect spectroscopy (ROESY)

2-*O*-Cin-α-CD and 3-*O*-Cin-α-CD are well soluble in water; therefore the supramolecular structures of both regioisomers were first investigated in D_2_O solutions. When the cinnamyl moiety is included in the CD cavity thus forming intermolecular complexes or self-included intramolecular species, the 2D ROESY spectrum shows this interaction as NOE crosspeaks between the inner hydrogens (H3 and H5) of the CD cavity and between the aromatic or double bond hydrogens of the cinnamyl moiety.

**Figure 2:**
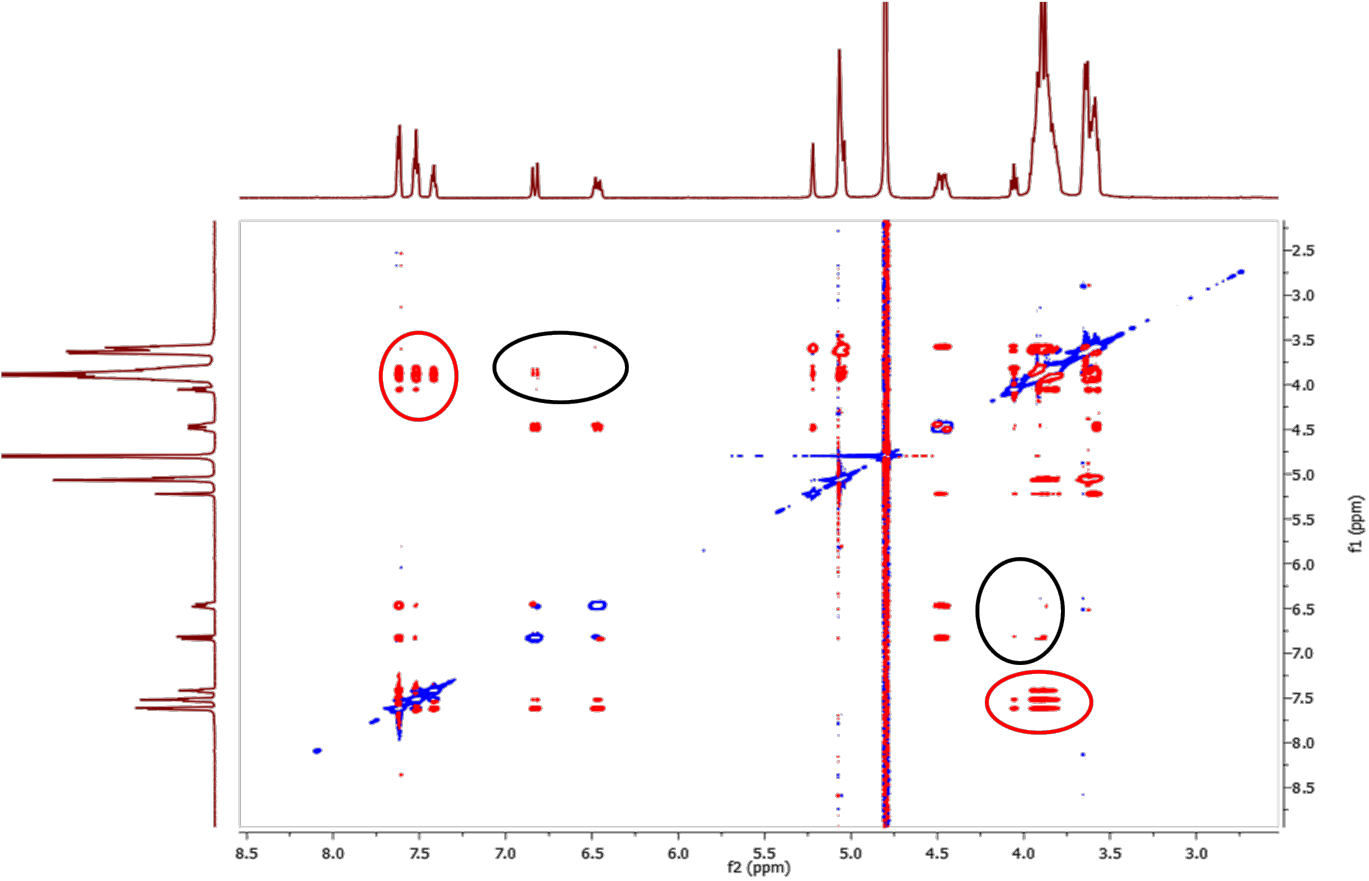
2D ROESY spectrum of 2-*O*-Cin-α-CD in D_2_O at 25 °C at 24 mM concentration.

In the spectra of both regioisomers intense correlations were observed between the phenyl protons (7.2–7.5 ppm) and the protons of the CD cavity (3.6–4.1 ppm) (red circles in Figure 2) proving that the phenyl part is located in the CD cavity.

**Figure 3:**
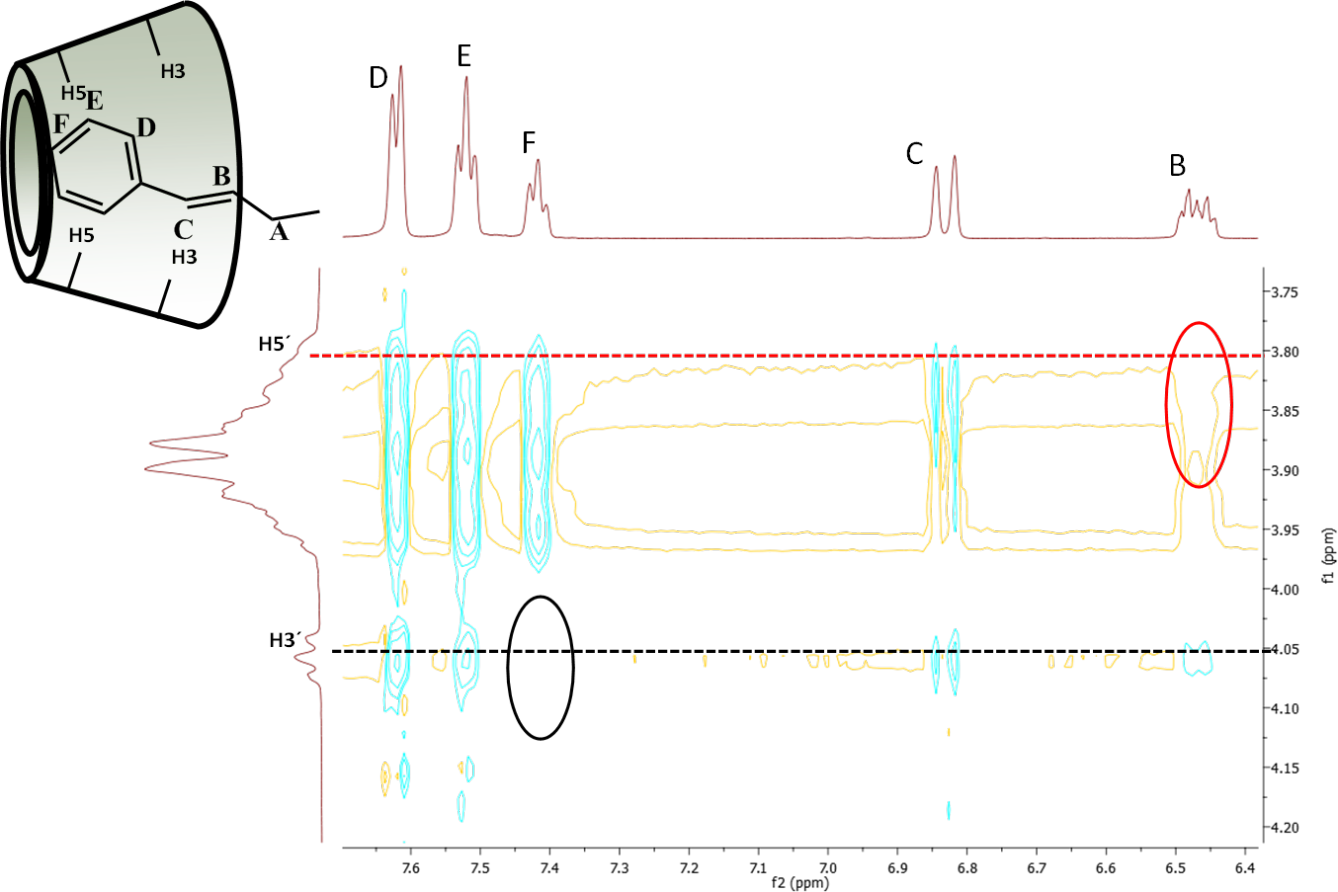
Expansion of the 2D ROESY spectrum of 2-*O*-Cin-α-CD indicating the geometric arrangement.

These correlations were observed for both inner proton region at 3.6–3.8 ppm for protons H5 (red dashed line in Figure 3) and 3.8–4.1 for protons H3 (black dashed line in Figure 3) indicating, that the cinnamyl moiety penetrated deeply into the CD cavity. Less intense cross-correlation was found for the two protons from the double bond region of the cinnamyl moiety (6.2–6.7 ppm, black circles in Figure 2), but their presence indicates that the double bond is also situated in the close proximity of the cavity and therefore contributing to the host−guest interaction. More detailed study of the ROESY spectra (see Figure 3) provided relevant information about the mode of the host–guest interaction.

Important differences can be observed between the interactions of the two double bond protons (B and C in Figure 3) with the cavity. In the 2D ROESY spectra of both regioisomers proton C interacts with both cavity protons (H3 and H5), indicating that proton C is penetrated into the cavity, on the other hand proton B shows interaction only with H3 but there is no interaction with H5 (red circle in Figure 3), suggesting that the linker in the host–guest complex is closer to the wider rim of the CD. The aromatic protons D, E and F of the isomer **2** also interact differently with the two inner protons of the cavity. While protons D and E show cross-correlation with both protons, proton F interacts only with H5 but it does not interact with proton H3 (black circle in Figure 3). These observations altogether leave us with only one possible mode of interaction, which is the inclusion of the cinnamyl moiety to the cavity from the secondary side (side close to the H3 protons) and not from the primary side (side close to the H5 protons) of the CD’s cavity.

The 2D ROESY spectra of the two regioisomers are comparable, (for 2D ROESY spectra of 3-*O*-Cin-α-CD see Figure SI 13 and Figure SI 14 in Supporting Information File 1) showing that in both cases the cinnamyl group is located inside the CD cavity and in both molecules this inclusion phenomenon takes place from the secondary side of the CD cavity.

### Supramolecular structures of 2-*O*-Cin-α-CD and 3-*O*-Cin-α-CD characterized by a ^1^H NMR dilution experiment

The observed NOE signals proved the close spatial proximity of the inner protons of the CD cavity and the aromatic and double bond protons of the cinnamyl moiety. However these experimental evidences do not unambiguously confirm the existence of the intermolecular interaction, because self-inclusion processes cannot be excluded. In order to prove our assumption that the NOE correlations arise from the intermolecular host–guest interactions, the systems were further investigated using a series of ^1^H NMR spectra recorded at different concentrations. The chemical shifts of ^1^H resonances of both regioisomers changed as a function of concentration, although those recorded in DMSO-*d*_6_ were concentration-independent.

**Figure 4:**
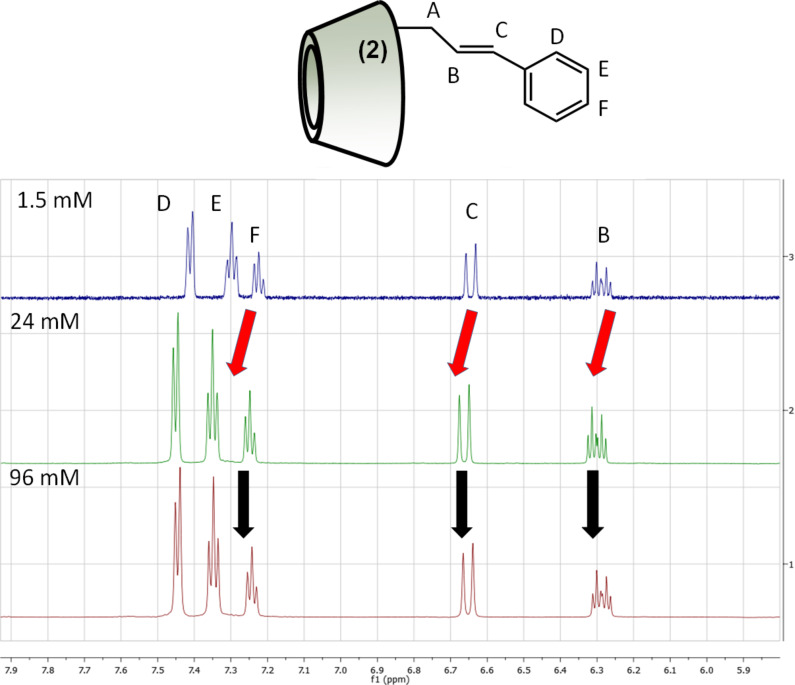
^1^H NMR spectra of 2-*O*-Cin-α-CD in D_2_O at 25 °C at different concentrations.

These results indicate that both regioisomers are capable forming intermolecular complexes in D_2_O solution, but they are in non-aggregated state in DMSO-*d*_6_. In the case of the isomer **2** downfield shifts in the aromatic and double bond region were observed when the concentration was changed gradually from 1.5 mM to 24 mM (Figure 4). Further increase in the concentration did not result in additional peak shifts, which means that the system stabilized at around 24 mM concentration and even if the concentration was increased dramatically (up to 96 mM) the chemical environment of the aromatic/double bond protons did not change significantly (Figure 4).

**Figure 5:**
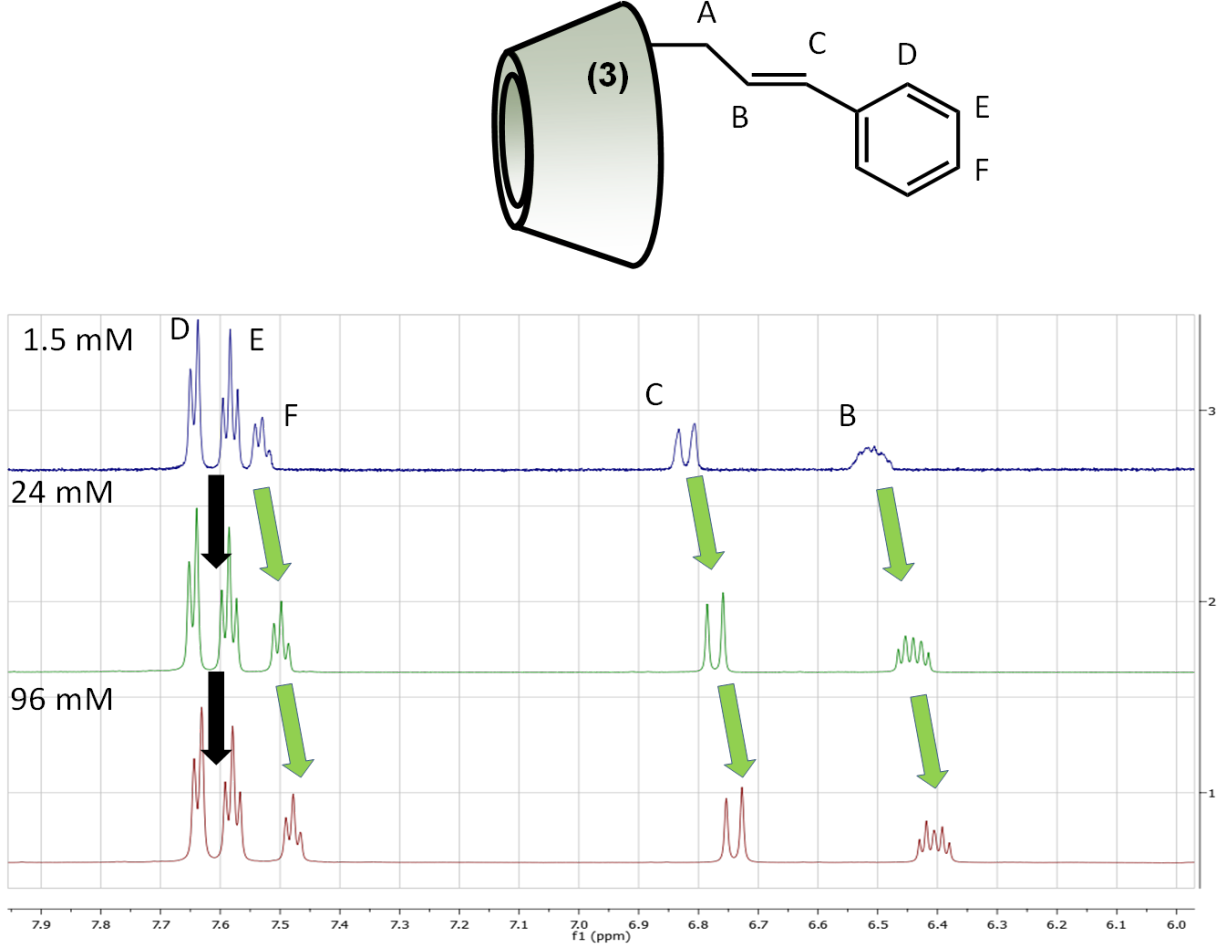
^1^H NMR spectra of 3-*O*-Cin-α-CD in D_2_O at 25 °C recorded at various concentrations.

The increasing concentration also influenced the ^1^H NMR spectrum of the isomer **3**, showing that the 3-*O* derivative also forms intermolecular complexes, but surprisingly the concentration change had an opposite effect compared to the isomer **2** (Figure 5). The double bond protons (protons B and C in Figure 5) and the resonances of the proton at the *para* position of the cinnamyl moiety (proton F in Figure 5) were monotonously shifted towards lower frequency and did not show any plateau when the concentration was increased gradually from 1.5 mM to 96 mM. It was also surprising that the the *orto* and *meta* protons (protons D and E), which showed intense correlations in the 2D ROESY spectra (Figure SI 13 and SI 14, Supporting Information File 1), did not exhibit concentration-dependent chemical shift differences (Figure 5).

In conclusion, the observed concentration-dependent chemical shift changes in the aromatic and double bond region unequivocally prove the intermolecular interactions in the case of both regioisomers and clearly show that these two regioisomers form supramolecular structures in a different manner.

### Size estimations of intermolecular inclusion complexes: pulse field gradient spin-echo NMR experiments (PFGSE NMR)

PFGSE NMR experiments were performed in order to estimate and compare the sizes of the supramolecular assemblies formed by the two regioisomers. This technique allows the quantification of the diffusion coefficient (*D*) of structures at various concentrations.

**Figure 6:**
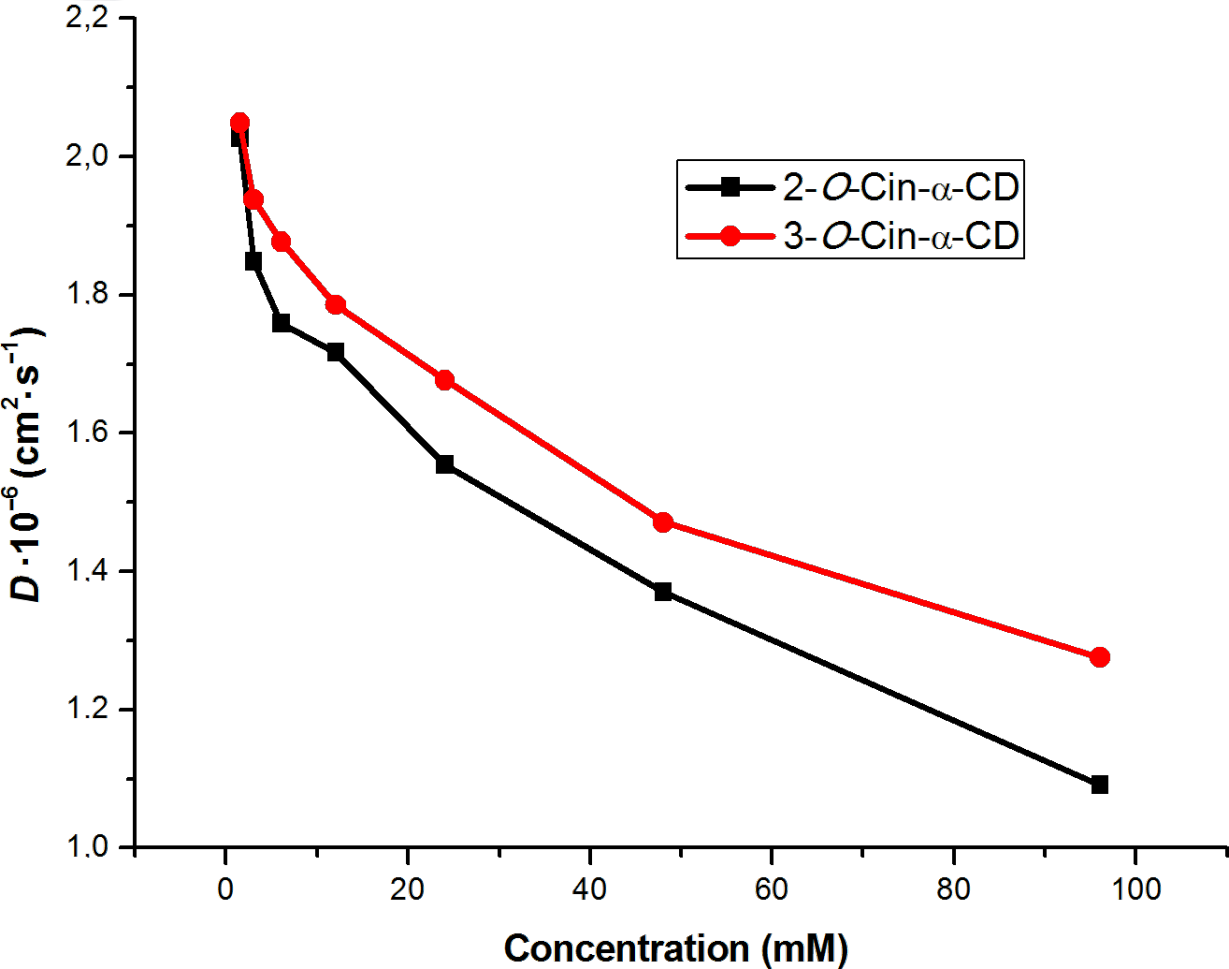
Diffusion coefficients of 2-*O*-Cin-α-CD (black) and, 3-*O*-Cin-α-CD (red) in D_2_O at various concentration at 25 °C.

The *D* of the unmodified α-CD was previously reported by Avram and Cohen [13] and it was found to be 3.0 × 10^-6^ cm^2^ s^−1^. The *D* of a supramolecular dimer formed by the structurally similar 2-*O*-Cio-α-CD was measured in work [9] and its value was found to be 2.3 × 10^−6^ cm^2^ s^−1^ at 10 mM concentration and did not change with further increase in the concentration. In the case of supramolecular polymerization the *D* should continuously decrease with increase in the concentration because of the increasing size of the supramolecular assembly. When the concentration of isomer **2** was gradually increased from 1.5 mM to 96 mM a monotonous decay in *D* was observed from 2.03 × 10^−6^ cm^2^ s^−1^ at 1.5 mM to 1.09 × 10^−6^ cm^2^ s^−1^ at 96 mM, respectively. The same concentration dependence was observed for the *D* of isomer **3** starting from 2.05 × 10^−6^ cm^2^ s^−1^ at 1.5 mM arriving to 1.27 × 10^−6^ cm^2^ s^−1^ at 96 mM (Figure 6).

These data indicate that the size of the supramolecular structures formed by the assembly of 2-*O*-Cin-α-CD or by 3-*O*-Cin-α-CD is apparently much larger than the size of the unmodified α-CD and also exceeds the size of the double threaded dimer formed by 2-*O*-Cio-α-CD. Furthermore we can conclude that because in the studied concentration range no stabilization of *D* was observed for none of the derivatives, these regioisomers form an opened supramolecular aggregate, which size in D_2_O increases with increase in the concentration.

### Size estimations of intermolecular inclusion complexes and other assemblies: DLS experiments

**Figure 7:**
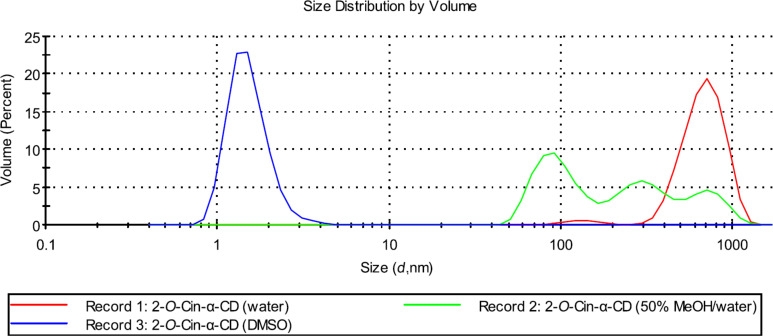
Effect of solvent on the size distribution of aggregates formed by 2-*O*-Cin-α-CD at 25 °C (the applied concentrations are 10 mg/mL (9.2 mM)).

**Figure 8:**
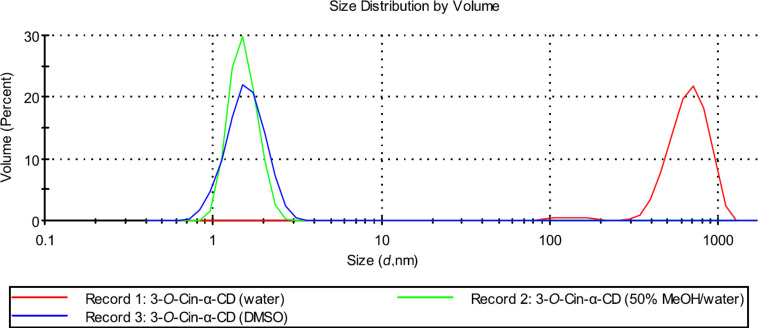
Effect of a solvent on the size distribution of aggregates formed by 3-*O*-Cin-α-CD at 25 °C (the applied concentrations are 10 mg/mL (9.2 mM)).

In order to further investigate the supramolecular structures and the effect of different external stimuli on the size distribution of the supramolecular assemblies a series of DLS experiments were performed. As a first parameter, the effect of different solvents on the size distribution was tested. In aqueous solution both regioisomers formed large aggregates with a hydrodynamic diameter (*D*_h_) up to 700 nm and the size of the aggregates formed by isomer **2** or isomer **3** were comparable (see Record 1 in Figure 7 and Figure 8).

In DMSO, no aggregation was observed for both derivatives (see Record 3 in Figure 7 and Figure 8). Those were present in molecularly dispersed form with a *D*_h_ around 1.25 nm, which corresponds to the size of the unmodified, non-aggregated α-CD [14]. These results are in agreement with the results obtained by NMR experiments, where no chemical shift changes were observed upon dilution in DMSO-*d*_6_ also indicating the absence of the intermolecular interactions.

Different aggregate sizes were observed for the two regioisomers in 50% MeOH solution (Record 2 in Figure 7 and Figure 8). Isomer **2** was present in a form of aggregates with a very broad size distribution ranging from 100 nm to 700 nm, while isomer **3** was found in a disaggregated form (*D*_h_ = 1.25 nm).

**Figure 9:**
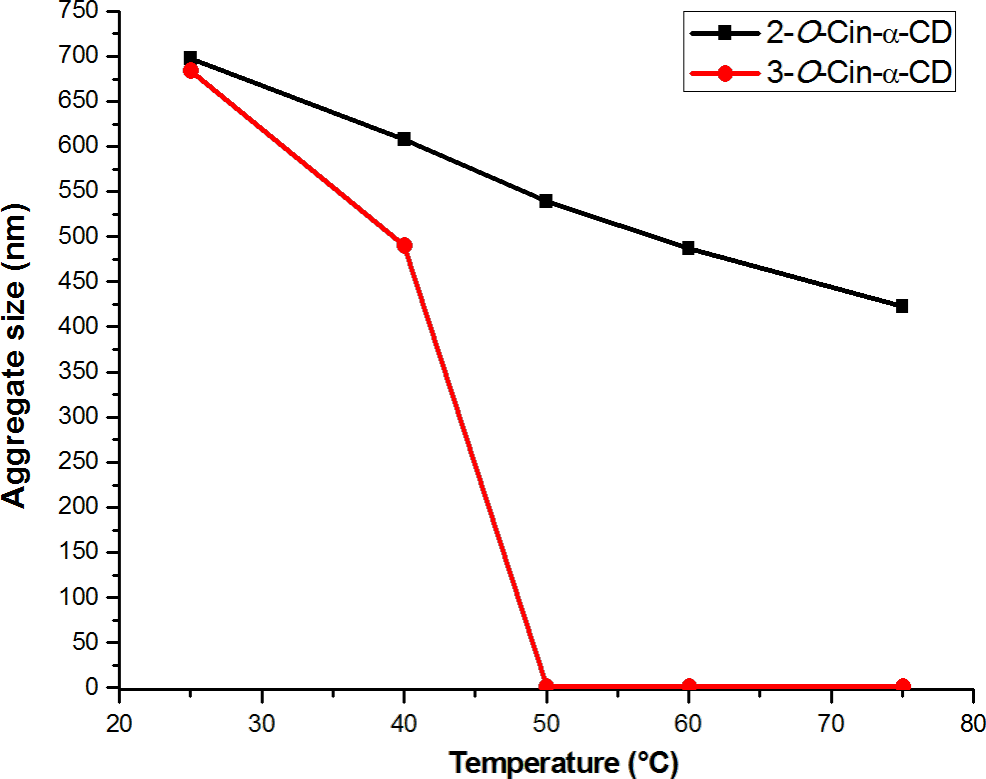
Aggregate sizes (diameter) of 2-*O*-Cin-α-CD (black) and 3-*O*-Cin-α-CD (red) in water at various temperatures (the applied concentrations are 10 mg/mL (9.2 mM)).

The temperature also influenced the size of the aggregates formed by the two regioisomers differently (Figure 9). Monotonous decay in size distribution was observed for the aggregates formed by the isomer **2** in water when the temperature was gradually elevated, although large aggregates with a *D*_h_ of 400 nm were still present at 75 °C.

On the other hand the aggregates formed by the isomer **3** in water were much more labile and they completely disaggregated as the temperature reached 50 °C. From these results we can conclude that the temperature can be used as an external stimulus to adjust the size distribution in water and to study its impact on the separation efficiency in separation techniques. The temperature-caused disaggregation is a reversible process – cooling back the solutions to a room temperature led to the formation of large particles with *D*_h_ comparable to the size of the initial particles.

The large aggregate sizes observed in water also indicate the formation of supramolecular structures, although it must be noted that only the host–guest interaction between the cinnamyl moiety and the α-CD cavity would not be strong enough to hold together such large aggregates. According to Boutellier [15] the degree of polymerization (*DP*_max_) for a SP is given by the association constant between the interacting components and by the concentration of the self-assembling monomer: *DP*_max_ = (*K* × *c*)^0.5^. The highest stability constants reported for the *trans*-cinnamyl derivatives with α-CD are on the order of 2 × 10^3^ M^−1^ [16]. If only this host–guest interaction would be involved in the intermolecular interaction, the degree of polymerization according to Boutellier’s model at 10 mg/mL (9.2 mM) concentration would give *DP*_max_ = 4.28, therefore the supramolecular chain should not be longer than 5 nm.

Apparently the *D*_h_ observed by DLS for both Cin-α-CD isomers (700 nm) is much larger than the calculated value (5 nm), which indicates that the SP formation is a much more complicated process and besides the host–guest interaction other additional intermolecular interactions may also take place. Due to the numerous hydroxy groups present in the studied molecules these secondary interactions most probably are the hydrogen-bond interactions between the CD units, or the π–π interactions of the cinnamyl moieties. The sum of all these possible interactions results in the large supramolecular aggregates observed by DLS.

### Effect of competitive additives on the aggregation behavior: DLS and ROESY experiments

In order to demonstrate that in the case of the Cin-α-CD the main force which initializes the aggregate formation is the host–guest interaction between the monomer units, a DLS experiment was set up in order to measure the aggregate size distribution after successive addition of potential competitive host or guest molecules. We decided to prove, that the size of the supramolecular assembly can be modulated by the addition of suitable chain growth inhibitors, which are able to displace the cinnamyl moiety from the neighboring CD cavity, or to shield the cinnamyl moiety from the cavity of the adjacent Cin-α-CD. Based on the previous observation that elevated temperature resulted in partial or complete disaggregation depending on the type of the regioisomer the following experiment was performed.

**Figure 10:**
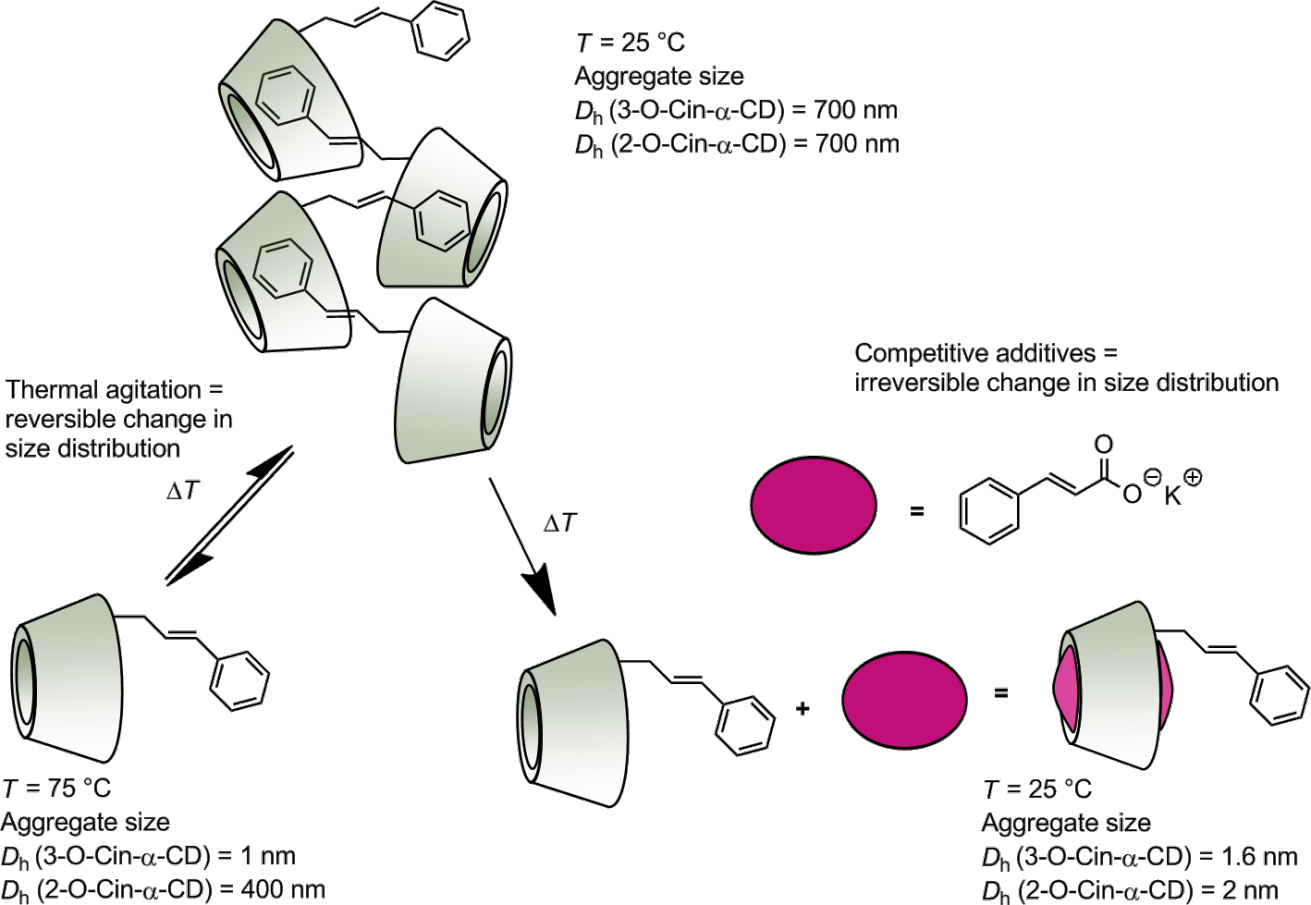
Schematic representation of the DLS experiment proving the host–guest nature of the aggregate formation.

The aqueous solution (10 mg/mL) of our derivatives was equilibrated at a temperature of 75 °C, stirred in a closed vial for 12 hours and then the system was perturbed by the addition of the inhibitor in a 10-fold molar excess respect to the Cin-α-CD. Subsequently the system was cooled back to room temperature and the size distribution of the aggregates was measured. In Figure 10 a schematic representation of the experiment setup is shown.

**Figure 11:**
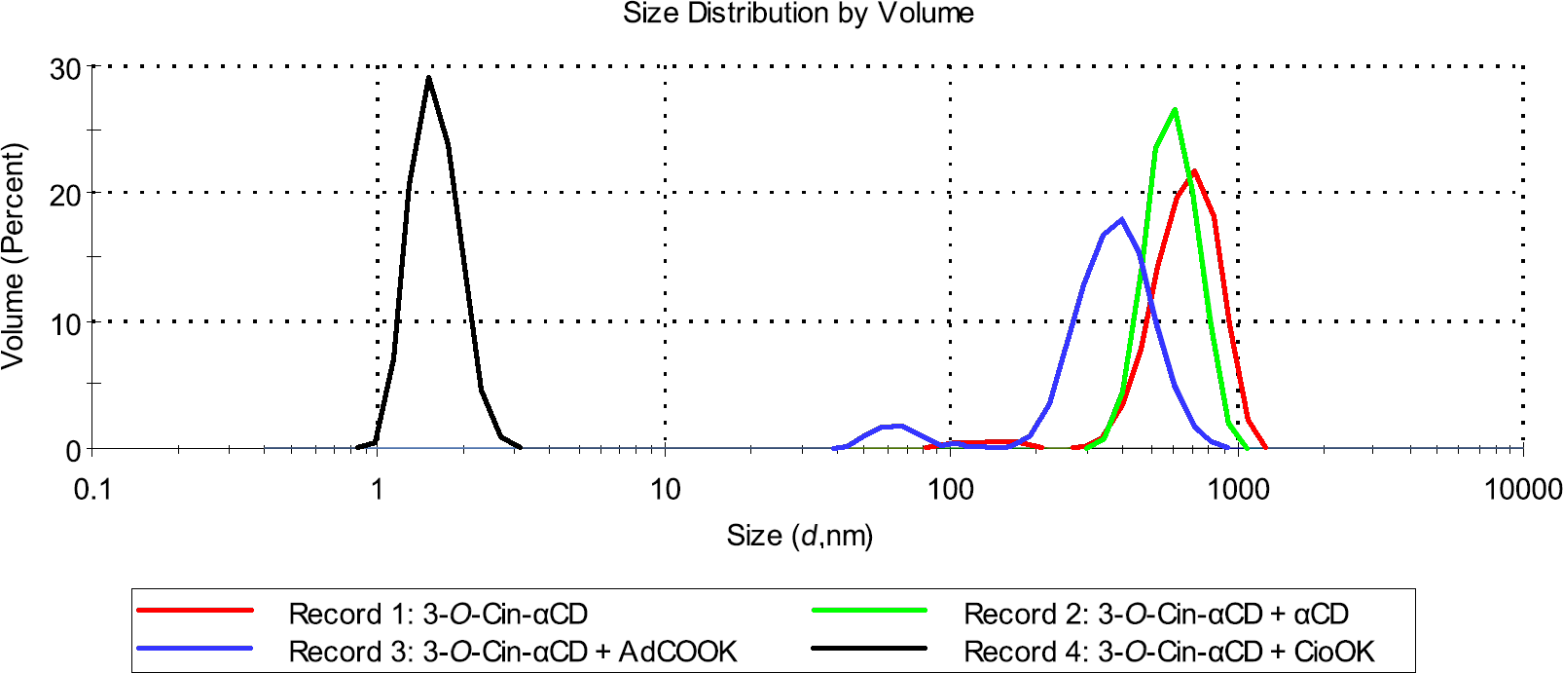
The effect of competitive additives on the size distribution of aggregates formed by 3-*O*-Cin-α-CD at 25 °C (the applied concentrations of the 3-*O*-Cin-α-CD are 10 mg/mL (9.2 mM)).

As shown in Figure 11 the addition of unmodified α-CD as competitive host molecule to the aqueous solution of isomer **3** did not decrease significantly the aggregate size distribution, which indicates that α-CD is not able to inhibit the intermolecular interactions between the molecules of 3-*O*-Cin-α-CD. Potassium adamantane-1-carboxylate (AdCOOK) was a more effective chain growth inhibitor as it decreased the aggregate size from 700 nm to 400 nm. Potassium cinnamate (CioOK) as a competitive guest molecule was able to completely displace the cinnamyl part of 3-*O*-Cin-α-CD from the adjacent CD cavity which resulted in decomposition of the host–guest complexes. As a result a significant decrease in the *D*_h_ of the aggregates (from 700 nm to 1.6 nm) was observed (Figure 10 and Figure 11).

The observed differences between the efficiency of the chain growth inhibition of AdCOOK and CioOK can be explained by the different size of the two competitive guest molecules. The α-CD cavity is able to accommodate the smaller CioOK, which results in a complete collapse of the supramolecular aggregates, on the other hand AdCOOK as a guest molecule is apparently too bulky for the α-CD cavity, therefore the displacement of the cinnamyl moieties in the intermolecular complexes and the caused disaggregation is only partial (from 700 nm to 400 nm). Isomer **2** showed a similar behavior upon addition of chain inhibitors as isomer **3** (Figure SI 15, Supporting Information File 1), which indicates that in case of both regioisomers the main force of the aggregation is the intermolecular host–guest interaction. The disrupted aggregates were stable at room temperature and did not show any aggregation in time, hence we can conclude that the disaggregation caused by competitive additives is an irreversible process.

Because CioOK as competitive additive showed remarkable changes in the aggregation behavior of Cin-α-CDs, we further investigated its interaction with both regioisomers using 1D and 2D NMR experiments to get a deeper insight into the disaggregation process. ROESY and ^1^H NMR spectra of isomer **2** or isomer **3** were recorded in the presence of CioOK (5-fold molar excess) in D_2_O after equilibrating the solutions at 75 °C and cooling back to 25 °C just before the measurements.

**Figure 12:**
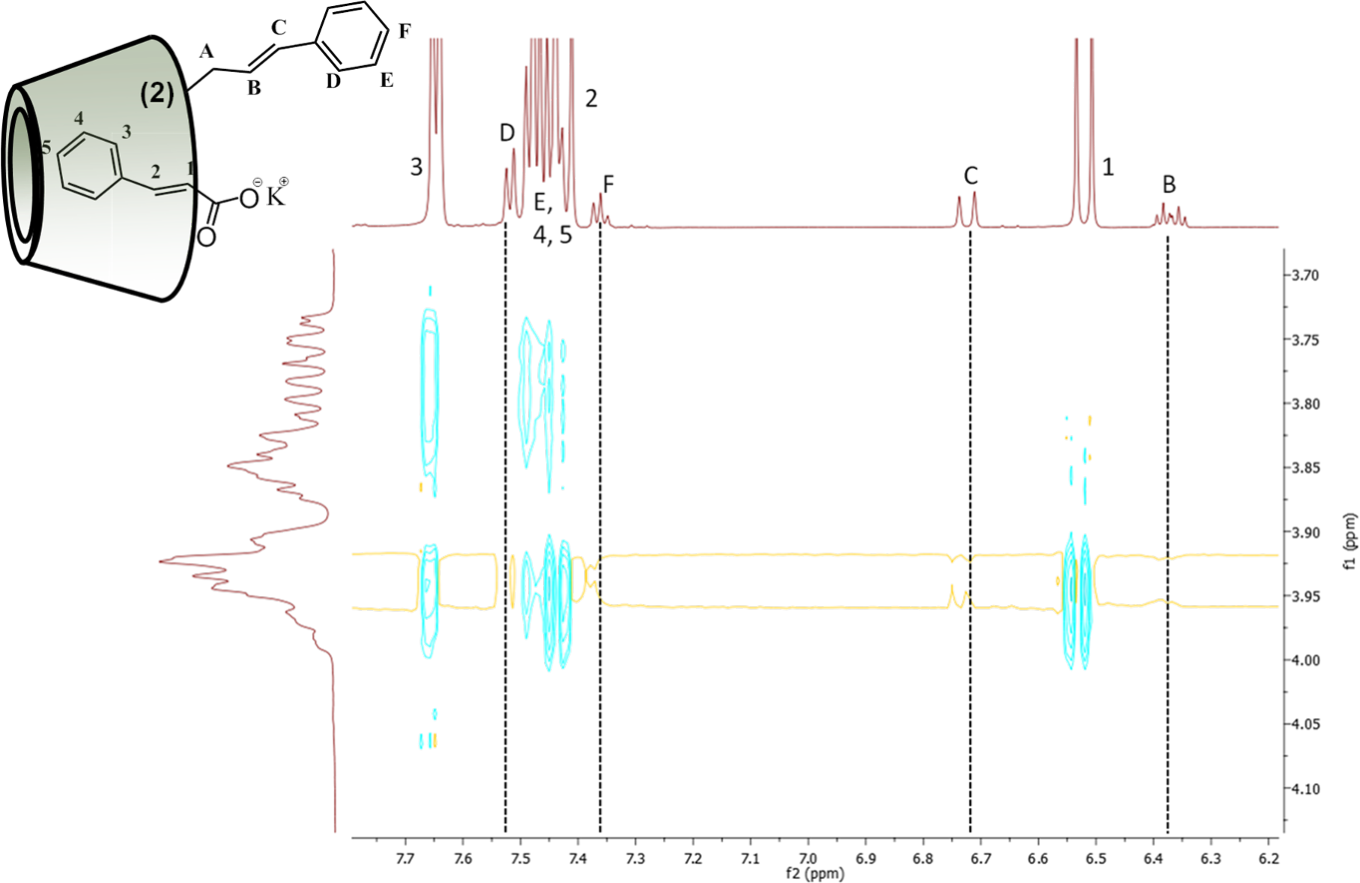
Expansion of the 2D ROESY spectrum of 2-*O*-Cin-α-CD in the presence of CioOK as competitive guest molecule in D_2_O at 25 °C.

Instead of the previously observed NOE cross-correlations in the ROESY spectra of both Cin-α-CD isomers, new cross-peaks appeared showing interaction only between potassium cinnamate and between the inner hydrogens of the CD cavity (Figure 12). The disappeared cross-peaks between the protons of the cinnamyl part of Cin-α-CDs (black dashed line in Figure 12) and between the CD cavity protons indicate that the cinnamyl moiety was effectively displaced by the additive.

**Figure 13:**
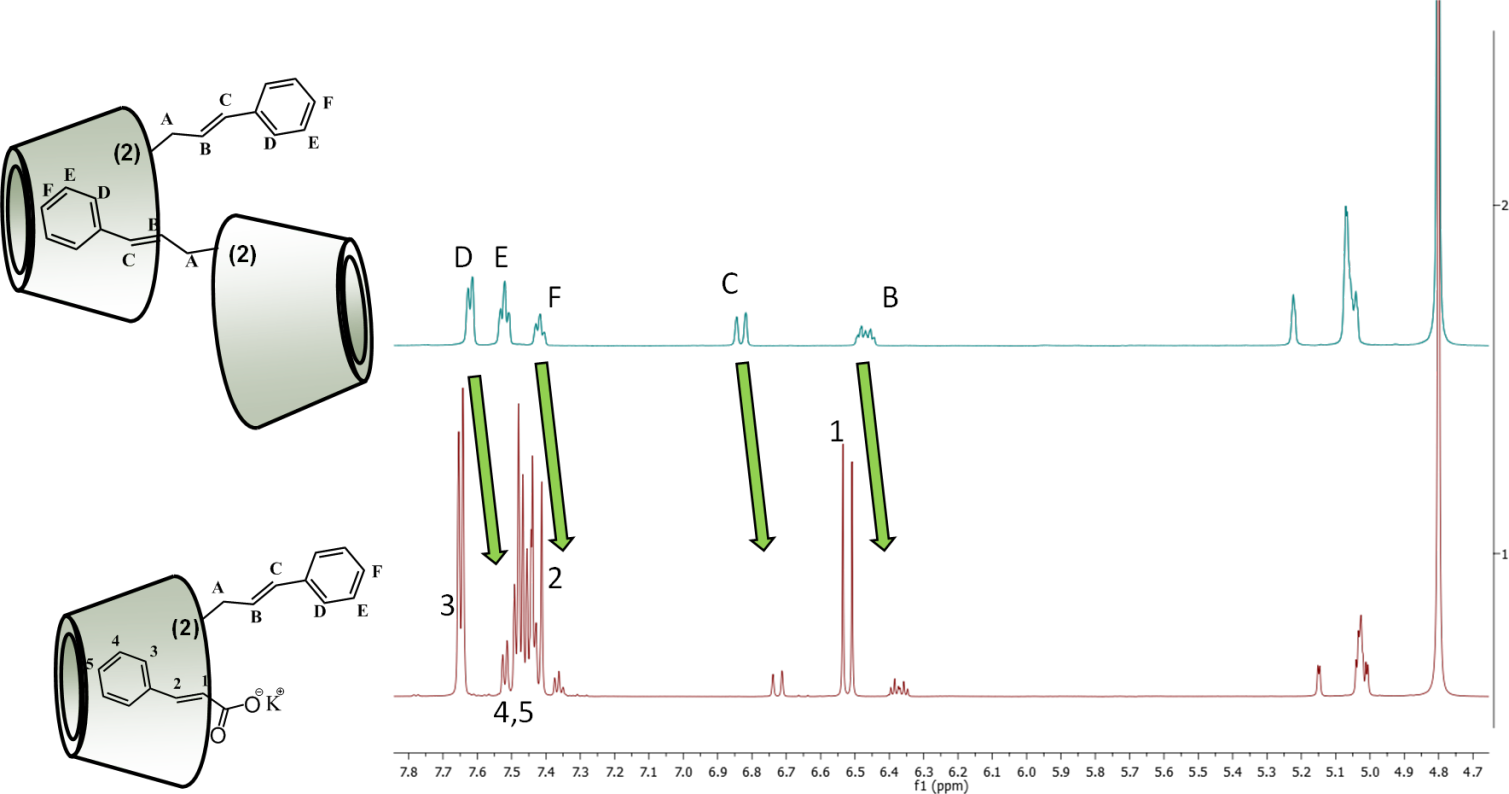
^1^H NMR spectrum of 2-*O*-Cin-α-CD before (up) and after (down) the addition of CioOK in 5-fold molar excess in D_2_O.

This phenomenon can be observed also in the ^1^H NMR spectrum of both isomers (Figure 13 for isomer **2**, Figure SI 21 in Supporting Information File 1, for isomer **3** respectively). If we compare the chemical shifts of aromatic or double bond protons of the given regioisomer before and after the addition of the competitive guest molecule, significant changes in chemical shifts can be detected, which indicates that the cinnamyl part of the molecule is located in a different chemical environment after the addition of the competitive guest.

These results are in a good agreement with the data obtained from DLS experiments and further confirmed our hypothesis which assumed that the observed disaggregation by CioOK is caused by the formation of a host–guest complex between Cin-α-CDs and between the molecules of CioOK.

### Analytical application

The potential of 2-*O*-Cin-α-CD and 3-*O*-Cin-α-CD as enantioselective agents was investigated by capillary electrophoresis. The influence of the addition of 2-*O*-Cin-α-CD and 3-*O*-Cin-α-CD to the background electrolyte (BGE) and its impact on the effective mobilities of eighteen selected analytes were tested. Nine analytes were in the form of cations (aniline, antipyrine, L-histidine, DL-tyrosine, DL-phenylalanine, *N*-(1-naphthyl)ethylenediamine, 4-nitroaniline, *p*-aminoacetophenone, tyramine) and nine in the form of anions (*N*-acetyl-DL-phenylalanine, *N*-acetyl-DL-tryptophan, *N*-benzoyl-DL-phenylalanine, *N*-boc-DL-tryptophan, *N*-FMOC-DL-valine, *N*-FMOC-DL-alanine, *N*-FMOC-DL-leucine, DL-3-phenyllactic acid, (*R*)-(−)-mandelic acid). Cations were separated in BGE consisted of 0.11 mol L^−1^ formic acid pH 2.3, while anions were separated in BGE with 10 mmol L^−1^ TRIS, pH 8.0 (pH was adjusted by formic acid). Because of the observation that the temperature differently influenced the size of the aggregates formed by Cin-α-CDs, the measurements were conducted at 25 and 50 °C. Both Cin-α-CD derivatives were added into BGE (pH 2.3 or 8.0) as aqueous solutions with final concentration of 5 or 25 mM. Differences between electrophoretic mobilities of the analytes were evaluated by two-tailed *t*-test.

**Figure 14:**
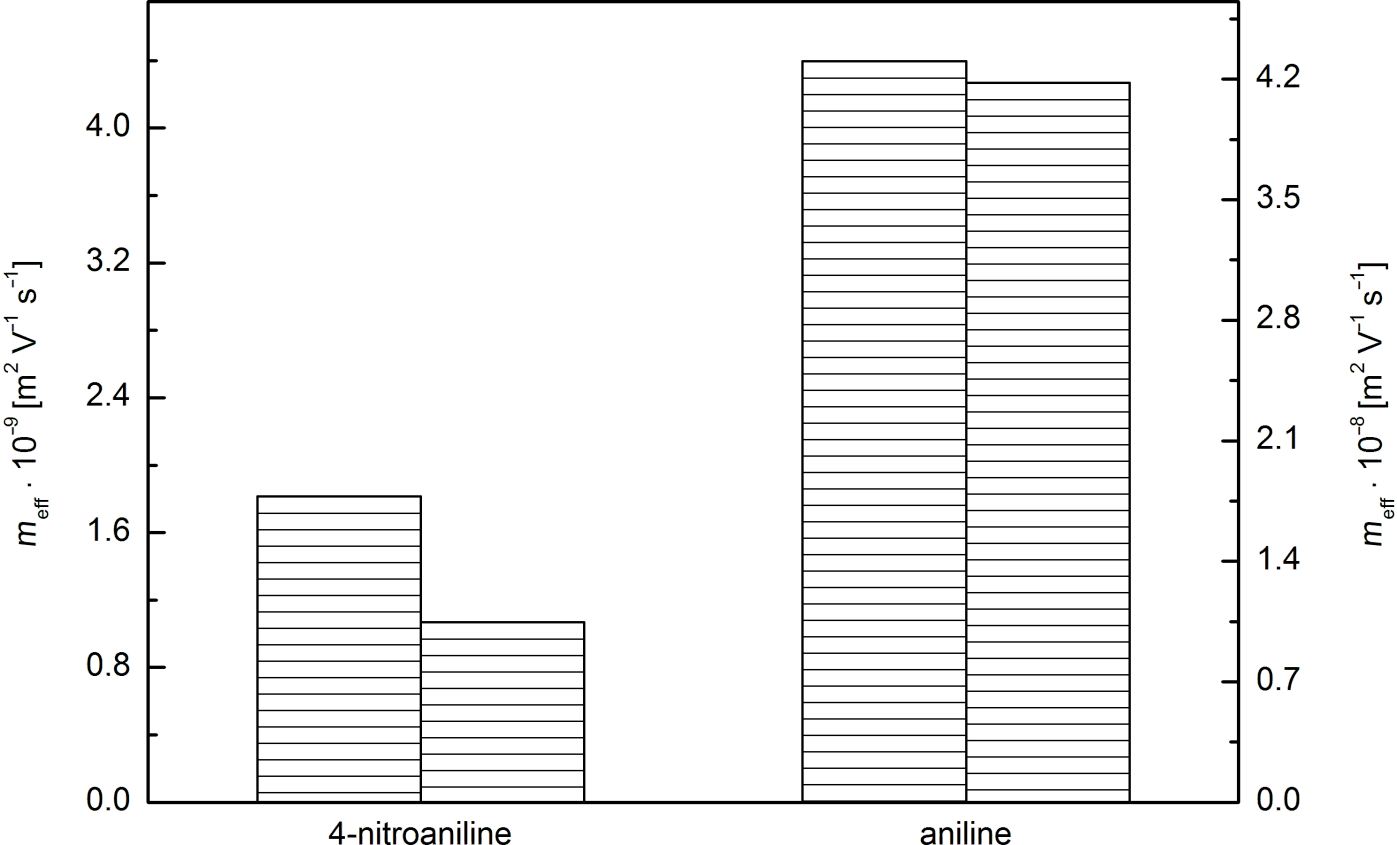
The influence of 5 mM 2-*O*-Cin-α-CD in BGE (right column) on the decrease of the effective electrophoretic mobility of 4-nitroaniline and aniline compared to the mobility in BGE without the additive (left column).

From all the tested cationic compounds a very significant decrease of the electrophoretic mobility was observed for 4-nitroaniline in the presence of 5 as well as 25 mM 2-*O*-Cin-α-CD in BGE, while electrophoretic mobility of aniline in the presence of 5 mM 2-*O*-Cin-α-CD did not change significantly. This phenomenon is an evidence for the important role of the nitro substituent in the structure of 4-nitroaniline in the interaction with the Cin-α-CD cavity (Figure 14).

*N*-(1-naphthyl)ethylenediamine significantly interacted with 5 mM 2-*O*-Cin-α-CD, however, the increase of the Cin-α-CD concentrations in BGE up to 25 mM caused stronger interactions with 3-*O*-Cin-α-CD in comparison with 2-*O*-Cin-α-CD. This fact indicates a specific interaction between *N*-(1-naphthyl)ethylenediamine and the 3-*O*-Cin-α-CD cavity.

Effective electrophoretic mobilities of all tested anions (listed above) did not decrease significantly in BGE with 5 mM Cin-α-CDs, although an increased concentration of Cin-α-CDs in BGE to 25 mM caused a decrease of the effective electrophoretic mobilities of all the above-mentioned analytes (cations as well as anions).

Measurements at 50 °C produced the expected results – electrophoretic mobilities of all analytes reached higher values due to lower viscosity of the BGE. Subsequently differences between electrophoretic mobilities with and without Cin-α-CDs in BGE decreased because of smaller size of Cin-α-CDs aggregates.

The studied α-cyclodextrin derivatives offer a potential for employing them in capillary electrophoresis as BGE additives because of their high solubility in aqueous solutions. Therefore, further studies of more advanced BGE additives containing guest and CD units are currently being performed.

## Conclusion

In summary, we have presented a straightforward approach towards the synthesis and characterization of two regioisomers of monocinnamyl-α-CD, which allowed their gram-scale preparation and their application as background electrolyte additives in capillary electrophoresis. A set of 2D NMR experiments were used to elucidate the structures of the prepared derivatives, thus to distinguish the two regioisomers. DLS and PFGSE NMR measurements were used to characterize the aggregation behavior of these derivatives and to show that both regioisomers are able to form highly-ordered polymeric structures through intermolecular interactions. The obtained results indicate that the size of these structures can be modulated by external stimuli, such as concentration, temperature or addition of guest molecules. Furthermore we showed that by using a suitable additive – potassium cinnamate as a competitive guest molecule, we can irreversibly inhibit the supramolecular aggregate formation.

## Supporting Information

File 1 General experimental procedures, instruments, materials. Detailed experimental procedures and characterization data for newly prepared compounds. Copies of ^1^H, ^13^C NMR, 2D NMR spectra of prepared compounds.
