# Supplementary material for: Supramolecular structures based on regioisomers of cinnamyl-α-cyclodextrins – new media for capillary separation techniques
Source: Beilstein J Org Chem. 2016 Jan 19;12:97–109. doi: 10.3762/bjoc.12.11 (PMC4734354; doi:10.3762/bjoc.12.11)
Supplement: File 1 — General experimental procedures, instruments, materials. Detailed experimental procedures and characterization data for newly prepared compounds. Copies of 1H, 13C NMR, 2D NMR spectra of prepared compounds. [file Beilstein_J_Org_Chem-12-97-s001.pdf]

Supporting Information

for

Supramolecular structures based on regioisomers of cinnamyl-α-cyclodextrins – new media for capillary separation techniques

Gabor Benkovics[1,2], Ondrej Hodek[3], Martina Havlikova[3], Zuzana Bosakova[3], Pavel Coufal[3], Milo Malanga[2], Eva Fenyvesi[2], Andras Darcsi[4], Szabolcs Beni[4] and Jindrich Jindrich[1*]

Address: [1]Department of Organic Chemistry, Faculty of Science, Charles University in Prague, Hlavova 8, 128 43, Prague 2, Czech Republic, [2]CycloLab, Cyclodextrin R&D Ltd, Budapest, H-1097 Illatos út 7, Hungary, [3]Department of Analytical Chemistry, Faculty of Science, Charles University in Prague, Hlavova 8, 128 43, Prague 2, Czech Republic and [4]Department of Pharmacognosy, Semmelweis University, Budapest, H-1085 Üllői út 26, Hungary

Email: Jindřich Jindřich - jindrich@natur.cuni.cz

*Corresponding author

General experimental procedures, instruments and materials. Detailed experimental procedures and characterization data for newly prepared compounds.
Copies of ^{1}H, ^{13}C NMR, 2D NMR spectra of prepared compounds

Contents

Instruments, general procedures and materials. S3

Synthesis of Cin-α-CD regioisomers. S5

Determination of diffusion coefficients. S6

References S6

2^{I}-*O*-Cinnamyl-α-cyclodextrin (2-*O*-Cin-α-CD) S7

Figure SI 1. ^{1}H NMR spectrum of 2-*O*-Cin-α-CD in D_2O.

Figure SI 2. ^{13}C NMR spectrum of 2-*O*-Cin-α-CD in D_2O.

Figure SI 3. DEPT-ed-HSQC spectrum of 2-*O*-Cin-α-CD (CD region) in D_2O.

Figure SI 4. HMBC spectrum of 2-*O*-Cin-α-CD (CD region) in D_2O as a proof of the substituent position.

Figure SI 5. COSY spectrum of 2-*O*-Cin-α-CD (CD region) in D_2O for the identification of H2' and H3'.

Figure SI 6. 1D TOCSY spectra of 2-*O*-Cin-α-CD (CD region) in D_2O for the identification of protons H3' and H5'.

3^{I}-*O*-Cinnamyl-α-cyclodextrin (3-*O*-Cin-α-CD) S14

Figure SI 7. ^{1}H NMR spectrum of 3-*O*-Cin-α-CD in D_2O.

Figure SI 8. ^{13}C NMR spectrum of 3-*O*-Cin-α-CD in D_2O.

Figure SI 9. DEPT-ed-HSQC spectrum of 3-*O*-Cin-α-CD (CD region) in D_2O.

Figure SI 10. HMBC spectrum of 3-*O*-Cin-α-CD (CD region) in D_2O as a proof of the substiuent position.

Figure SI 11. COSY spectrum of 3-*O*-Cin-α-CD (CD region) in D_2O for the identification of proton H2' and H3'.

Figure SI 12. 1D TOCSY spectra of 3-*O*-Cin-α-CD (CD region) in D_2O for the identification of protons H3 and H5.

Figure SI 13. 2D ROESY spectrum of 3-*O*-Cin-α-CD in D_2O at 25 °C at 24 mM concentration.

Figure SI 14. Expansion of the 2D ROESY spectrum of 3-*O*-Cin-α-CD indicating the geometric arrangement.

Figure SI 15. Effect of competitive host and guest molecules on the size distribution of the 2-*O*-Cin-α-CD (the applied concentrations of the 2-*O*-Cin-α-CD are 10 mg/mL (9.2 mM)).

Figure SI 16. Autocorrelation functions for DLS experiment: Effect of solvent on the size distribution of aggregates formed by 2-*O*-Cin-α-CD at 25 °C

Figure SI 17. Autocorrelation functions for DLS experiment: Effect of solvent on the size distribution of aggregates formed by 3-*O*-Cin-α-CD at 25 °C

Figure SI 18: Autocorrelation functions for DLS experiment: Effect of competitive host and guest molecules on the size distribution of aggregates formed by 2-*O*-Cin-α-CD at 25 °C.

Figure SI 19: Autocorrelation functions for DLS experiment: Effect of competitive host and guest molecules on the size distribution of aggregates formed by 3-*O*-Cin-α-CD at 25 °C.

Figure SI 20: ^{1}H NMR spectrum of CioOK in D_2O at 25 °C.

Figure SI 21: ^{1}H NMR spectrum of 3-*O*-Cin-α-CD before (up) and after (down) the addition of CioOK in 5-fold molar excess in D_2O

Instruments, general procedures and materials

α-CD was purchased from CycloLab R&D Laboratory, cinnamyl bromide (97% purity), *trans*-cinnamic acid, adamantane-1-carboxylic acid and sodium hydride (60% dispersion in mineral oil) was purchased from Sigma-Aldrich. All the other reagents and materials were purchased from commercial sources and used without further purification**.** Formic acid, 99%, 4-nitroaniline, 99%, and 4-amino-2,3-dimethyl-1-phenyl-3-pyrazolin-5-one, 98%, were purchased from Merck. D,L-phenylalanine, 99%, *N*-acetyl-L-phenylalanine, 99%, *N*-acetyl-

D-phenylalanine, ≥99.0%, *N*-boc-L-tryptophane, 99%, *N*-boc-D-tryptophane, ≥ 98.0%, *N*-benzoyl-L-phenylalanine, ≥99.0%, *N*-benzoyl-D-phenylalanine, ≥99.0%, D,L-phenyllactic acid, 98%, L-(−)-3-phenyllactic acid, ≥99.0%, (*R*)-(−)-mandelic acid, 98%, *N*-boc-D,L-phenylalanine, ≥99,0%, *N*-acetyl-L-tryptohane, ≥99.0%, *N*-acetyl-D,L-tryptophane, ≥ 99,0%, D,L-tyrosine, 99%, *N*-(1-naphtyl)ethylenediamine, ≥98%, L-histidin, ≥98%, *p*-aminoacetophenone, 99%, D,L-tyramine, ≥98%, aniline, ≥99%, L-phenylalanine, ≥98%, D,L-phenylalanine, 99%, *N*-FMOC-L-valin, ≥98%, *N*-FMOC-D-valin, ≥98%, *N*-FMOC-L-leucin, ≥97%, *N*-FMOC-D-leucin, ≥95%, *N*-FMOC-L-alanin, 95%, and *N*-FMOC-D-alanin, ≥98%, were purchased from Sigma-Aldrich. Sodium hydroxide, p.a., was purchased from Lach-Ner (Czech Republic) and tris(hydroxymethyl)aminomethan, p.a., was purchased from Lachema (Czech Republic).

^{1}H NMR spectra were acquired on Varian VXR-600 at 600 MHz, using the residual solvent signal as internal reference. Assignments were aided by COSY, DEPT-ed-HSQC, ROESY, 1D TOCSY and HMBC experiments.

Mass spectra were obtained on a Bruker ESQUIRE 3000 ES-ion trap instrument with electrospray ionization (ESI) in positive mode. All samples were dissolved in water.

Thin layer chromatography (TLC) was performed on silica gel coated aluminium sheets DC-Alufolien Keisegel 60 F265 (Merck, Darmstadt, Germany). Dipping in 50% H_2SO_4 with subsequent carbonization, a heat gun was used for spot detection of all CD derivatives.

Chromatographic separation of Cin-α-CD isomers was performed on a Büchi preparative chromatograph using SiliCycle SiliaCartridger – 40 mm Cartridge packed with Lichroprep RP-18 Phase (40–63 µm) reversed phase silica as a stationary phase, water–methanol gradient elution and Büchi UV Photometer C-635 as a detector (detection wavelength 226 nm).

Dynamic Light Scattering measurements were carried out on Malvern Zetasizer Nano ZS, UK instrument. Triplicate measurements were carried out for all samples, each averaged of at least 10 runs. The volume size distribution was utilized for aggregate state analysis.

Capillary electrophoresis measurements were performed on a G7100A – 7100 Capillary Electrophoresis Instrument with UV–vis detector (Agilent Technologies, USA). Every analyte was measured three times. A fused-silica capillary, 50 μm i.d. and 375 μm o.d. (CACO, Slovakia) of total length 50 cm (41.5 to the detection window) was used and analytes were detected at 200, 214 and 254 nm. Data were evaluated with ChemStation software (Agilent Technologies, USA). Sonication of standard solutions was provided by using the ultrasonic instrument LC30H (Elma, Czech Republic). The pH of background electrolyte was adjusted to desired value utilizing pH meter Jenway 3540 (Jenway, United Kingdom).

Synthesis of Cin-α-CD regioisomers

Dried α-CD (5 g, 5.14 mmol) was dissolved in dry DMSO (50 mL), and sodium hydride (60% dispersion in mineral oil) was added (0.148 g, 6.16 mmol) to the solution. The reaction mixture was stirred under an argon atmosphere for 3 h, then cinnamyl bromide (1.21 g, 6.16 mmol) was added to the reaction mixture. The reaction was monitored by direct-phase TLC (*n*-PrOH/H_2O/EtOAc/NH_4OH (25%) 6/3/1/1 v/v/v/v) and by reversed-phase TLC (H_2O/MeOH 1/1 v/v) and was determined as finished after 3 h when no significant increase in monosubstituted derivatives was observed. The reaction mixture was neutralized with HCl and then concentrated under reduced pressure at 70 °C. Addition of acetone (500 mL) to the concentrated DMSO solution yielded a white precipitate that was filtered and washed with acetone (3 × 50 mL). Direct-phase TLC showed that the precipitate consisted of a mixture of unreacted α-CD, monosubstituted α-CD and disubstituted α-CD, while the mother liquor contained only the unreacted cinnamyl bromide. The precipitate was dissolved in DMF (30%

solution) and injected to the preparative reversed-phase chromatographic column. The unreacted α-CD, the pure isomers of the mono-Cin-α-CD and the fraction of di-Cin-α-CD derivatives were eluted separately from the column using a gradient of water/methanol (from 100/0 to 50/50) elution mixture. Fractions with 90/10 (water/methanol) eluent were concentrated to give the 3-O-Cin-α-CD (3) in (1.05 g, 21% yield). Evaporation of fractions with the 70/30 (water/methanol) elution mixture afforded the 2-O-Cin-α-CD (0.45 g, 9% yield).

Determination of diffusion coefficients

The pulse field gradient spin-echo (PFGSE) NMR spectra were recorded at 600 MHz in D_2O on a Varian VXR-600 spectrometer at 25 °C. The bipolar pulse pair stimulated echo (BPPSTE) sequence was applied for PFGSE NMR measurement. The pulsed gradients' strength was increased from $6.36 \cdot 10^{-1}$ to 43.1 (gauss/cm). The time separation of pulsed field gradients and their duration were 0.10 and $1.0 \cdot 10^{-3}$ (s). The shape of the gradient pulse was rectangular and its strength varied automatically during the course of the experiments. The *D* values were determined from the slope of the regression line ln(*I/Io*) versus *G*2, according to the Stejskal and Tanner's Equation[1].

References

1. Stejskal, E. O.; Tanner, J. E. *J. Chem. Phys*. 1965, *42*, 288-292.

2^{I}-*O*-Cinnamyl-α-cyclodextrin (2-*O*-Cin-α-CD)

Atom numbering for ^{1}H NMR and for ^{13}C NMR

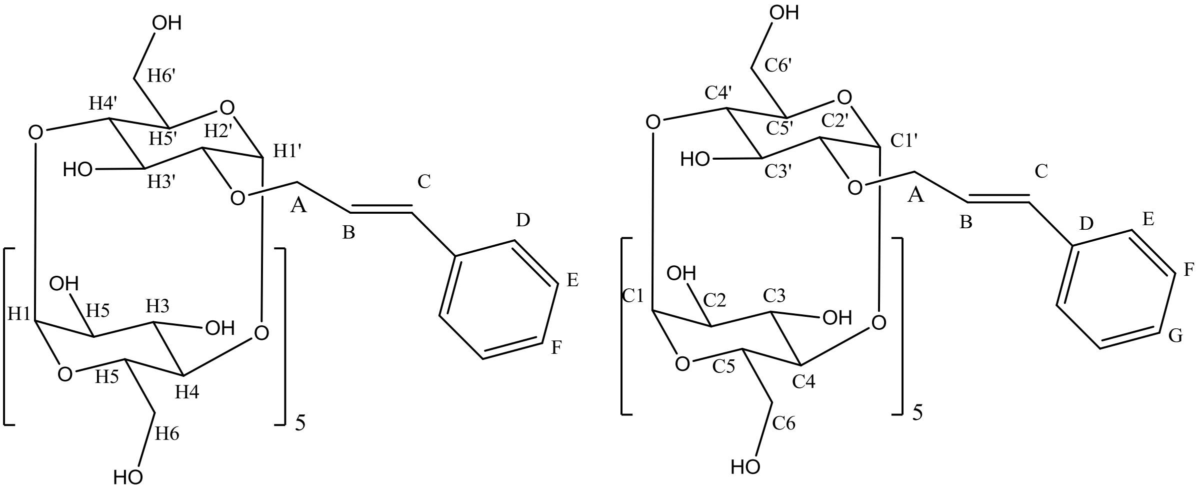

^{1}H NMR (600 MHz, D_2O) δ 7.62 (d, J = 7.5 Hz, 2H, D), 7.50 (dd, J = 7.3 Hz 2H, E), 7.41 (t, J = 7.5 Hz, 1H, F), 6.83 (d, J = 15.9 Hz, 1H, C), 6.50-6.42 (m, 1H, B), 5.22 (s, 1H, H1'), 5.12-4.99 (5H, H1), 4.47 (m, 2H, A), 4.05 (t,1H, H3'), 4.09-3.57 (m, 35H, H2, H3, H4, H5, H6, H2', H4', H5', H6').

^{13}C NMR (151 MHz, D_2O) δ 138.95 (D), 136.35 (C), 131.49 (F), 130.68 (G), 129.26 (E), 127.80 (B), 104.27–103.90 (C1), 102.16 (C1'), 84.55 – 73.90 (C2, C3, C4, C5, C2', C3', C4', C5'), 75.21 (C3'), 74.82 (A), 63.11-62.79 (C6), 63.00 (C6').

ESIMS: $[M+Na]^{+}$, found 1111.4. $C_{45}H_{68}O_{30}Na$ calculated 1111.3688

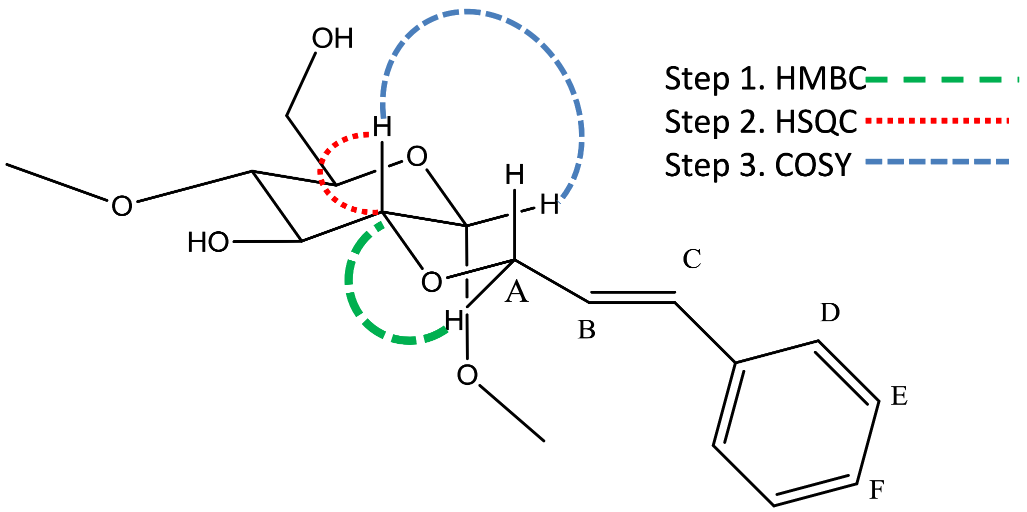

Elucidation:

Step 1. HMBC: H A (4.47 ppm) - C2’ (81.38 ppm) (Fig. SI 4)

Step 2. HSQC: C2’ (81.38 ppm) – H2’ (3.57 ppm) (Fig. SI 3)

Step 3. COSY: H2’ (3.57 ppm) - H1’ (5.22 ppm) (Fig. SI 5)

Verification:

No correlation in HMBC between C3’ (75.35 ppm) and proton A (4.47 ppm) (Figure SI 4)

No correlation in HMBC between C6’ (63.00 ppm) and proton A (4.47ppm) (Figure SI 4)

Conclusion:

Cinnamyl moiety is attached to the 2-*O*-position.

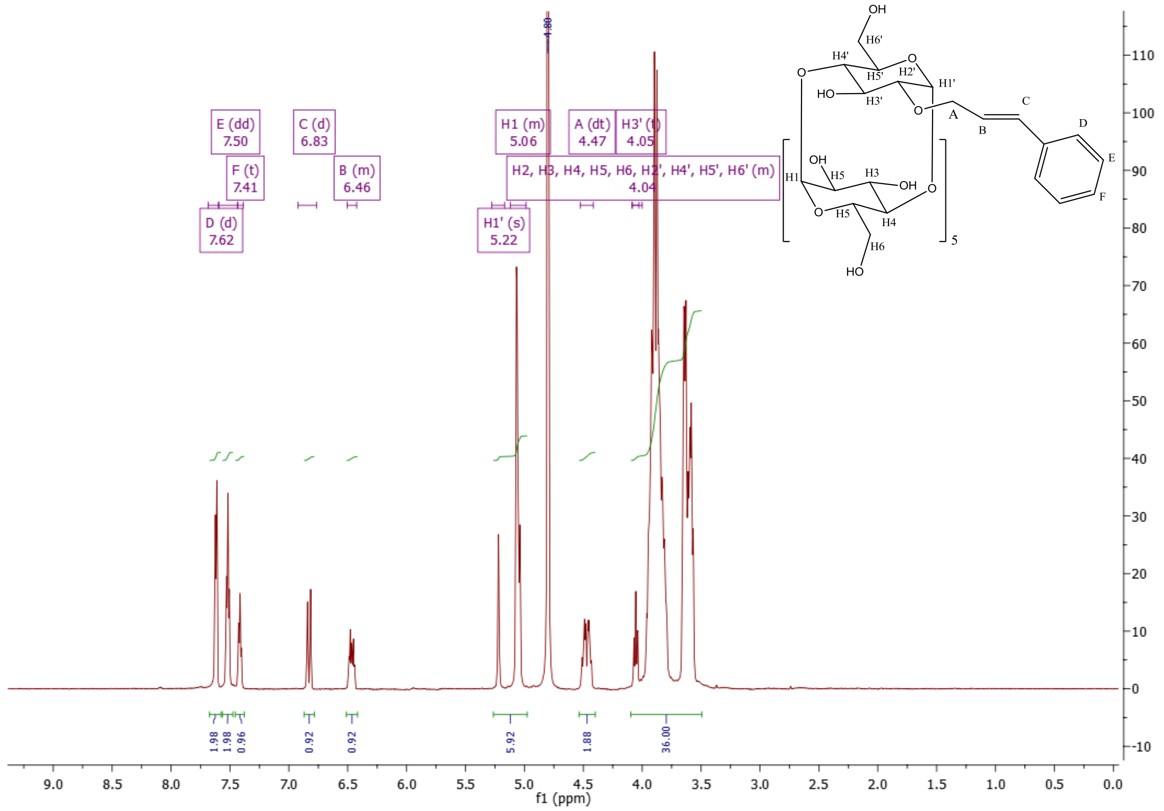

Figure SI 1: ^{1}H NMR spectrum of 2-*O*-Cin-α-CD in D_2O.

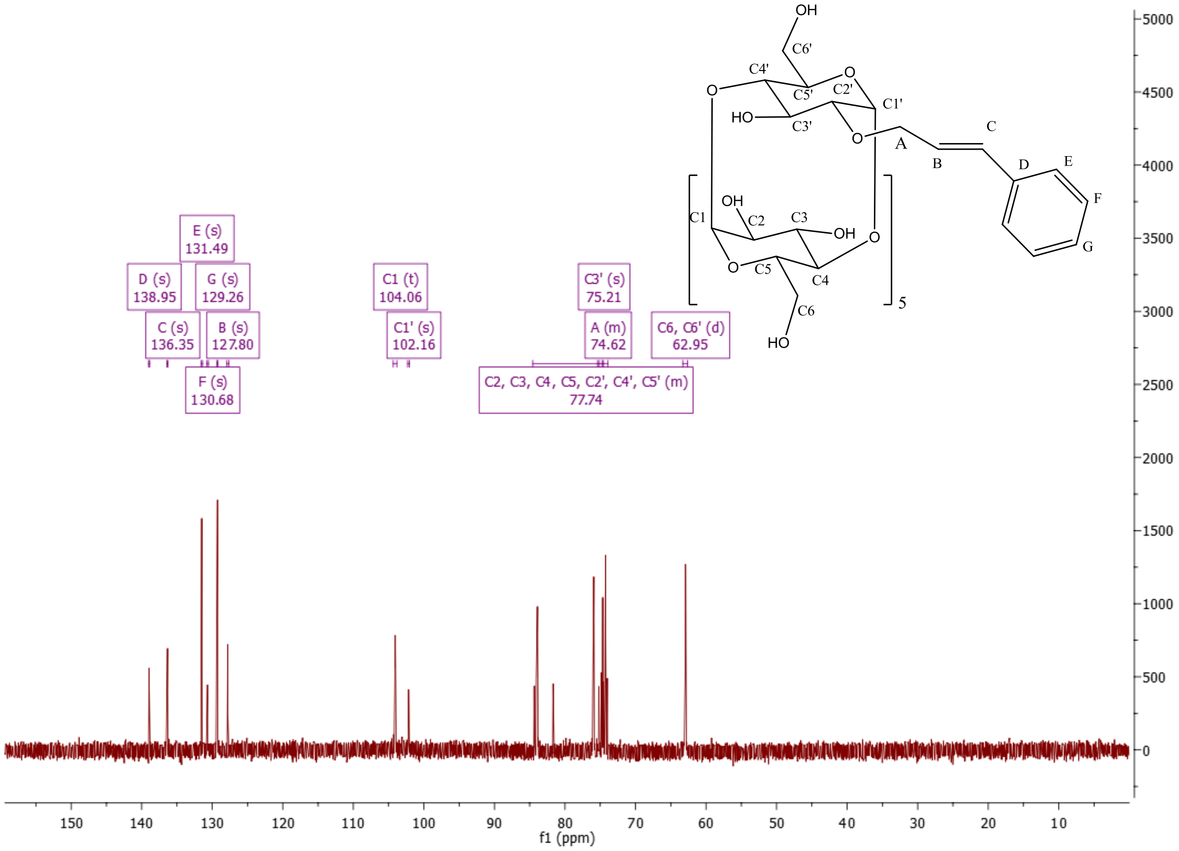

Figure SI 2:^{13}C NMR spectrum of 2-*O*-Cin-α-CD in D_2O.

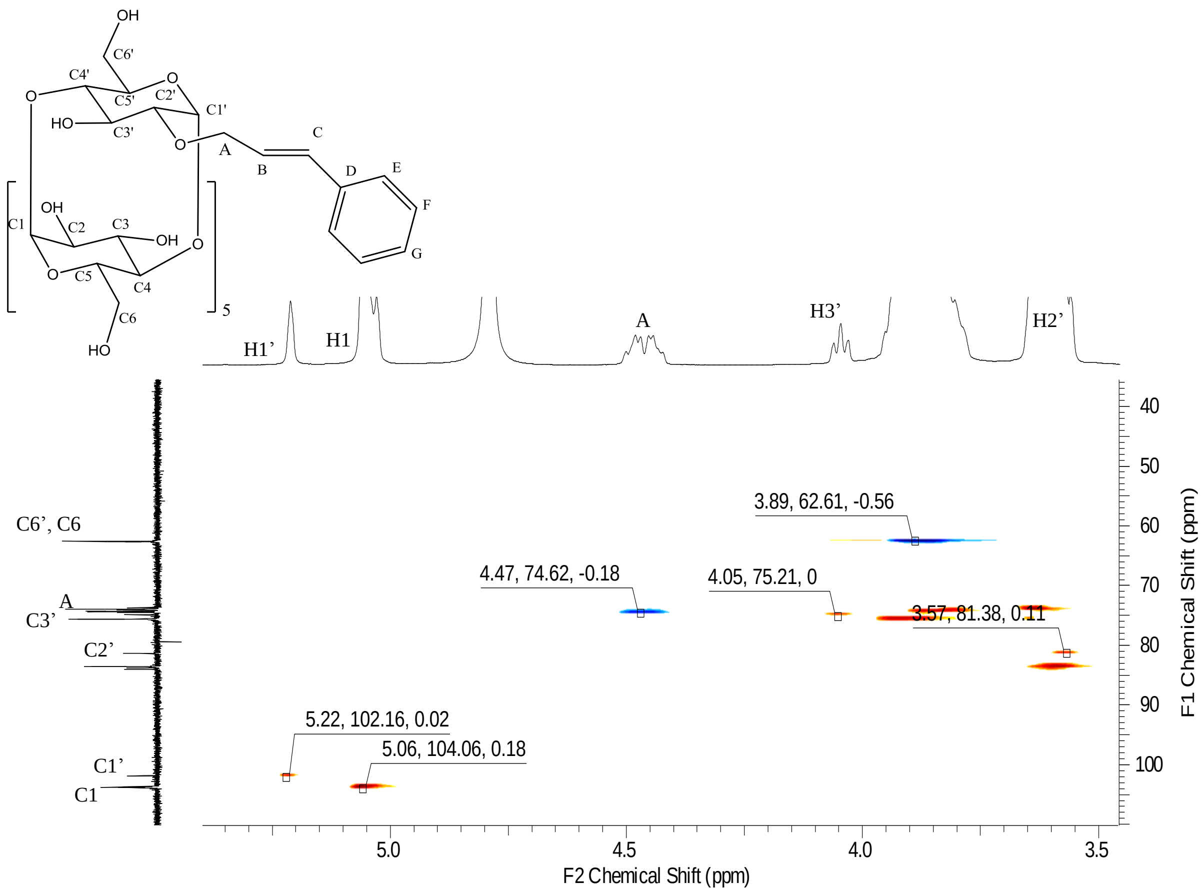

Figure SI 3: HSQC DEPT spectrum of 2-*O*-Cin-α-CD (CD region) in D_2O.

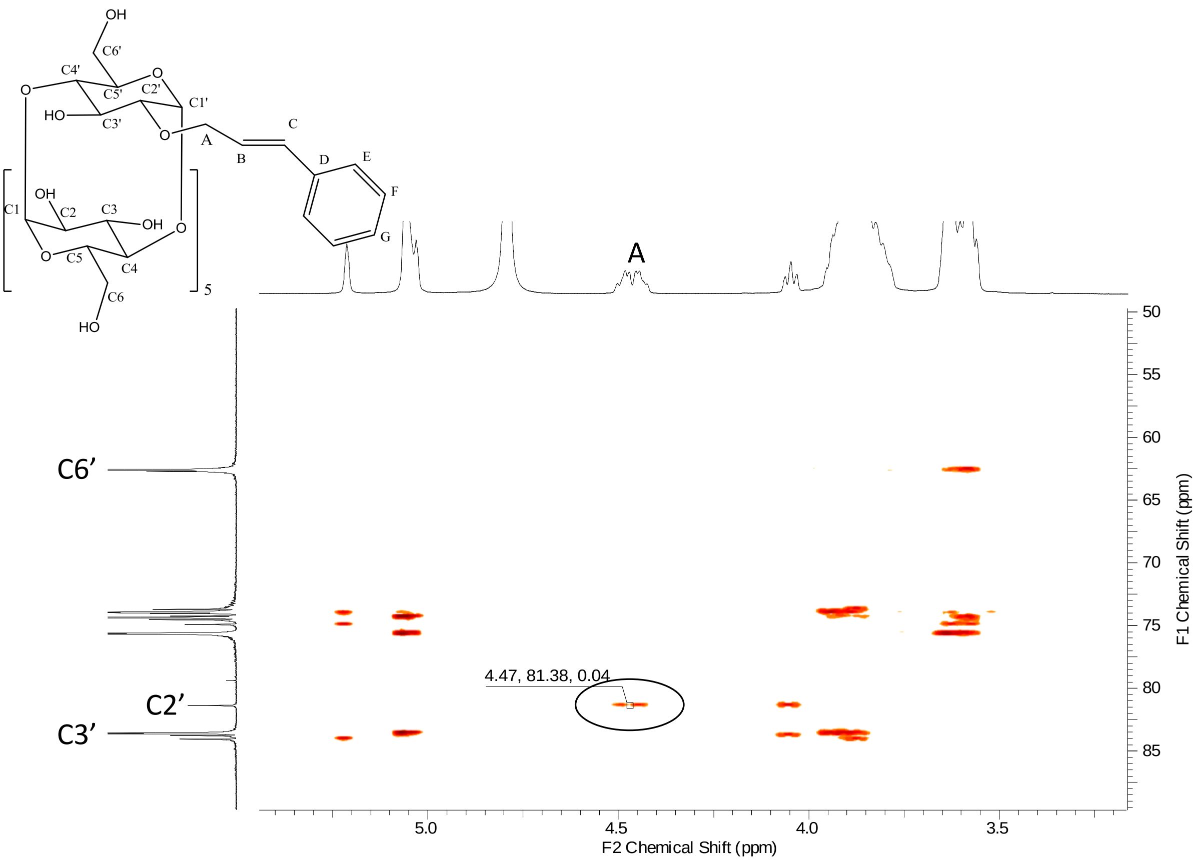

Figure SI 4: HMBC spectrum of 2-*O*-Cin-α-CD (CD region) in D_2O as a proof of the substiuent position.

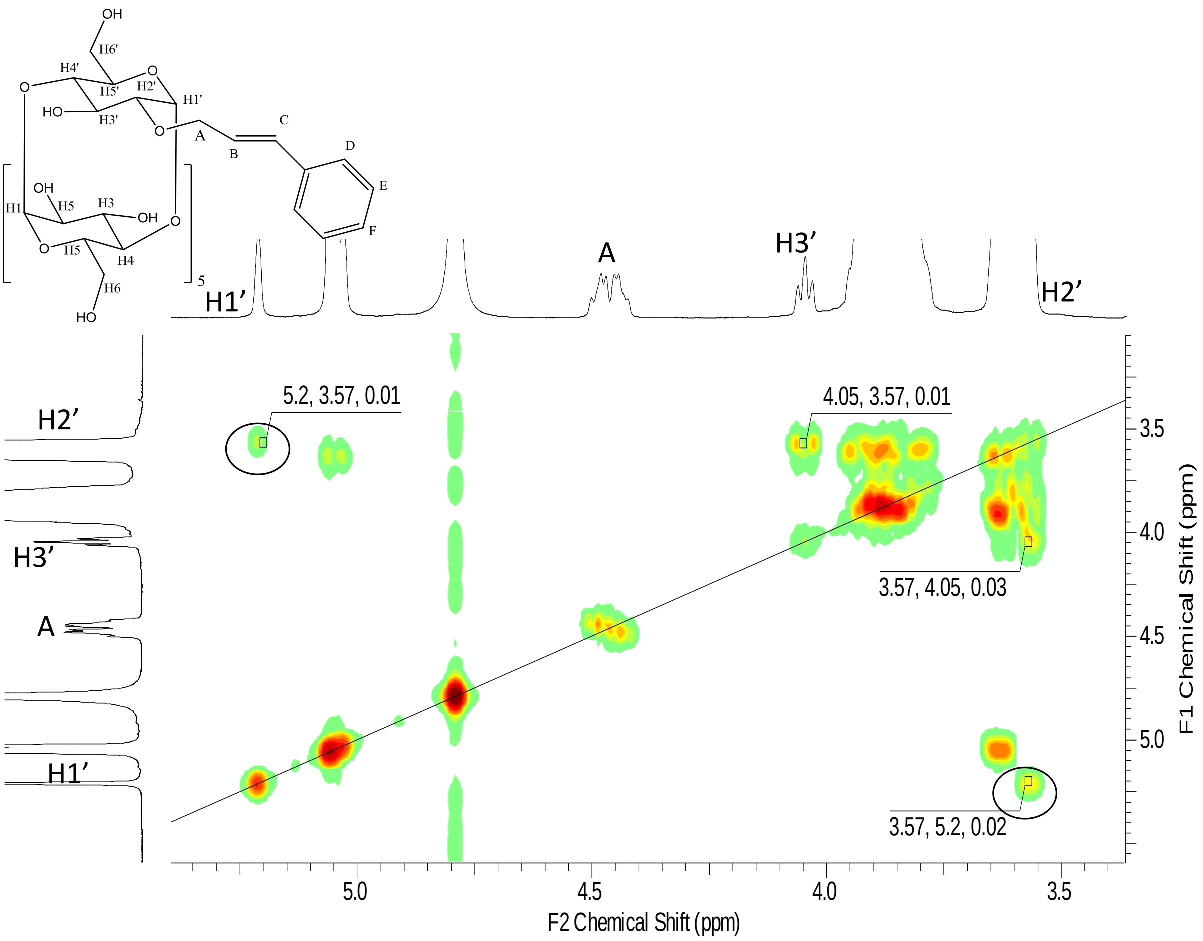

Figure SI 5: COSY spectrum of 2-*O*-Cin-α-CD (CD region) in D_2O for the identification of protons H2' and H3'.

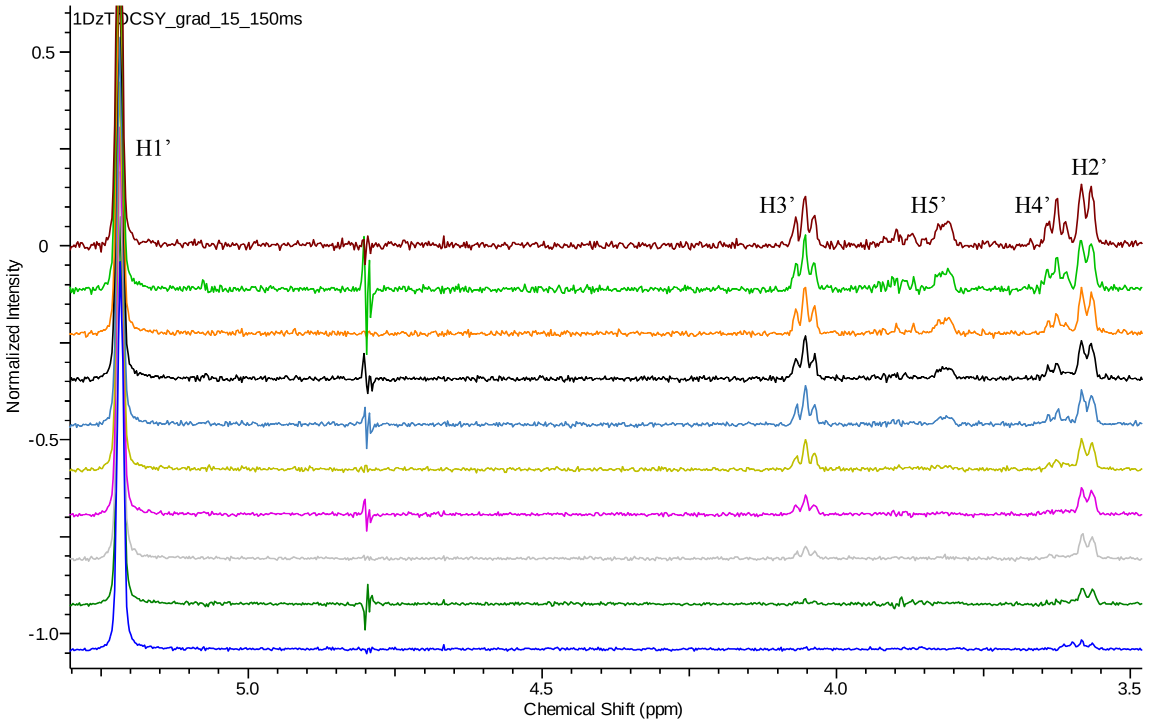

Figure SI 6: 1D TOCSY spectra of 2-*O*-Cin-α-CD (CD region) in D_2O for the identification of protons H3' and H5'.

3^{I}-*O*-Cinnamyl-α-cyclodextrin (3-*O*-Cin-α-CD)

Atom numbering for ^{1}H NMR and for ^{13}C NMR

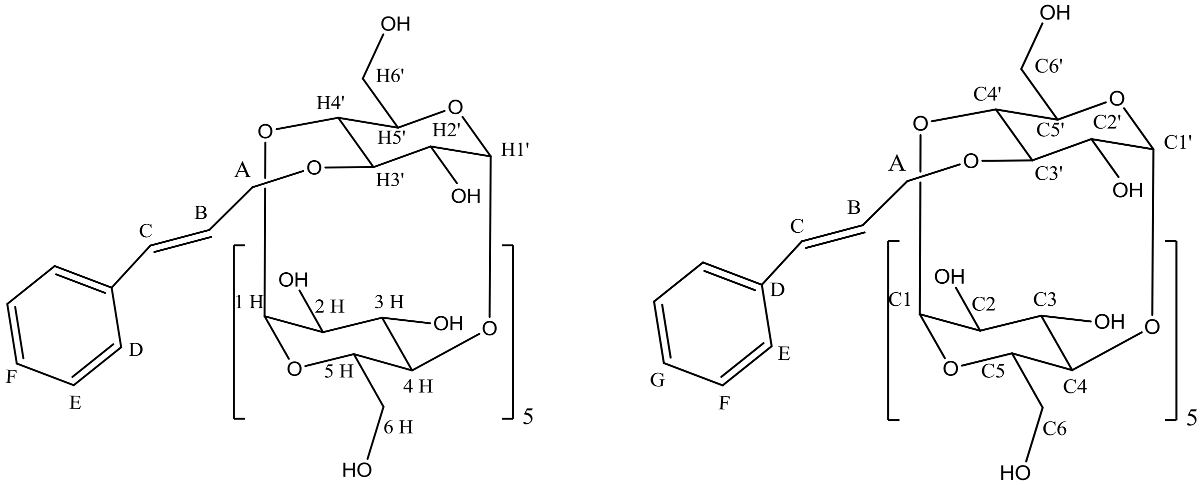

^{1}H NMR (600 MHz, D_2O) δ 7.63 (d, *J* = 7.6 Hz, 2H, D), 7.57 (dd, 2H, E), 7.50 (t, *J* = 7.3 Hz, 1H, F), 6.77 (d, *J* = 15.8 Hz, 1H, C), 6.48 – 6.40 (1H, B), 5.15 – 4.98 (6H, H1, H1'), 4.57 – 4.40 (m, 2H, A), 4.02 – 3.53 (m, 36H, H2, H3, H4, H5, H6, H2', H3', H4', H5', H6').

^{13}C NMR (151 MHz, D_2O) δ 138.98 (D), 138.44 (C), 131.64 (F), 130.94 (G), 129.16 (E), 127.22 (B), 104.72 – 103.49 (C1', C1), 84.31 – 83.65 (C4, C4'), 82.09 (C2'), 79.82 – 79.60 (C3'), 76.16 (A), 76.61 – 73.88 (C2, C3, C5', C5), 63.02-62.79 (C6, C6'). ESIMS: $[M+Na]^{+}$, found 1111.4. $C_{45}H_{68}O_{30}Na$ calculated 1111.3688

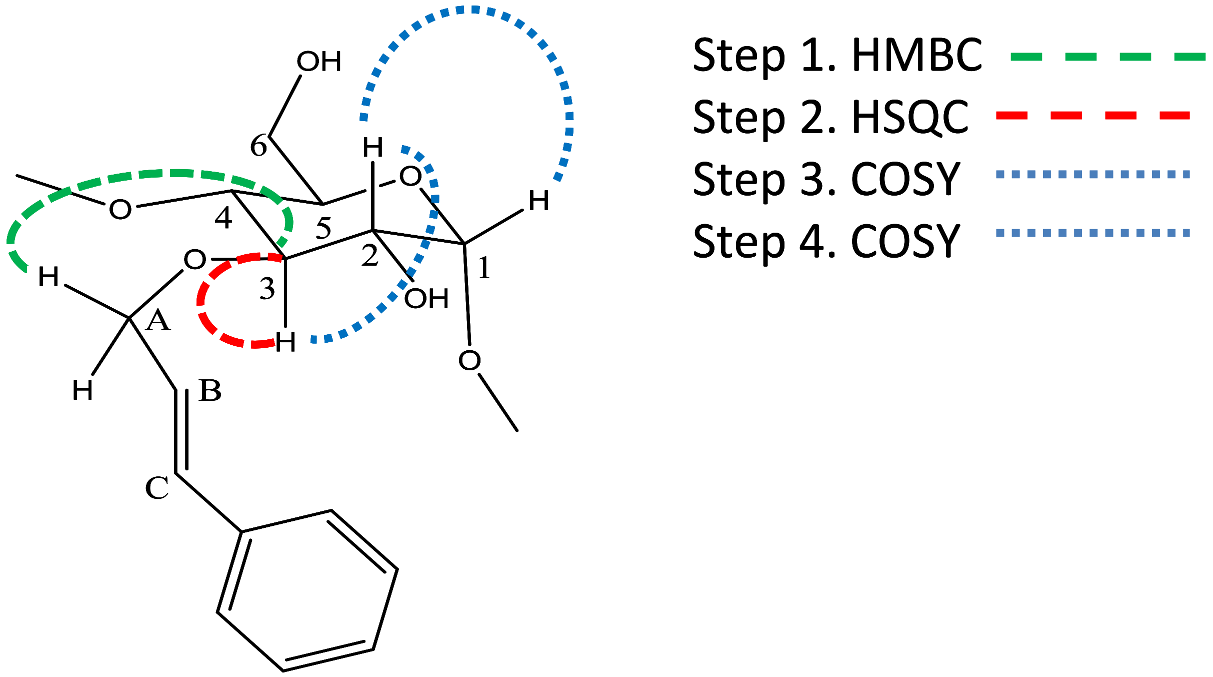

Elucidation:

Step 1. HMBC: H A (4.53 ppm) - C3’ (79.71 ppm) (Fig. SI 10)
Step 2. HSQC: C3’ (79.71 ppm) - H3’ (3.81 ppm) (Fig. SI 9)
Step 3. COSY: H3’ (3.81 ppm) – H2’ (3.62 ppm) (Fig. SI 11)
Step 4. COSY: H2’ (3.62 ppm) – H1’ (5.01 ppm) (Fig. SI 11)

Verification:

No correlation in HMBC between carbon C2’ (82.09 ppm) and proton A (4.33 ppm) (Fig. SI 9)
No correlation in HMBC between carbon C6’ (63.02-62.79 ppm) and proton A (4.33ppm) (Fig. SI 9)
No correlation in COSY between proton H3’ (3.81 ppm) and proton H1’ (5.01 ppm) (Fig. SI 11)

Conclusion:
Cinnamyl moiety is attached to the 3-*O*-position.

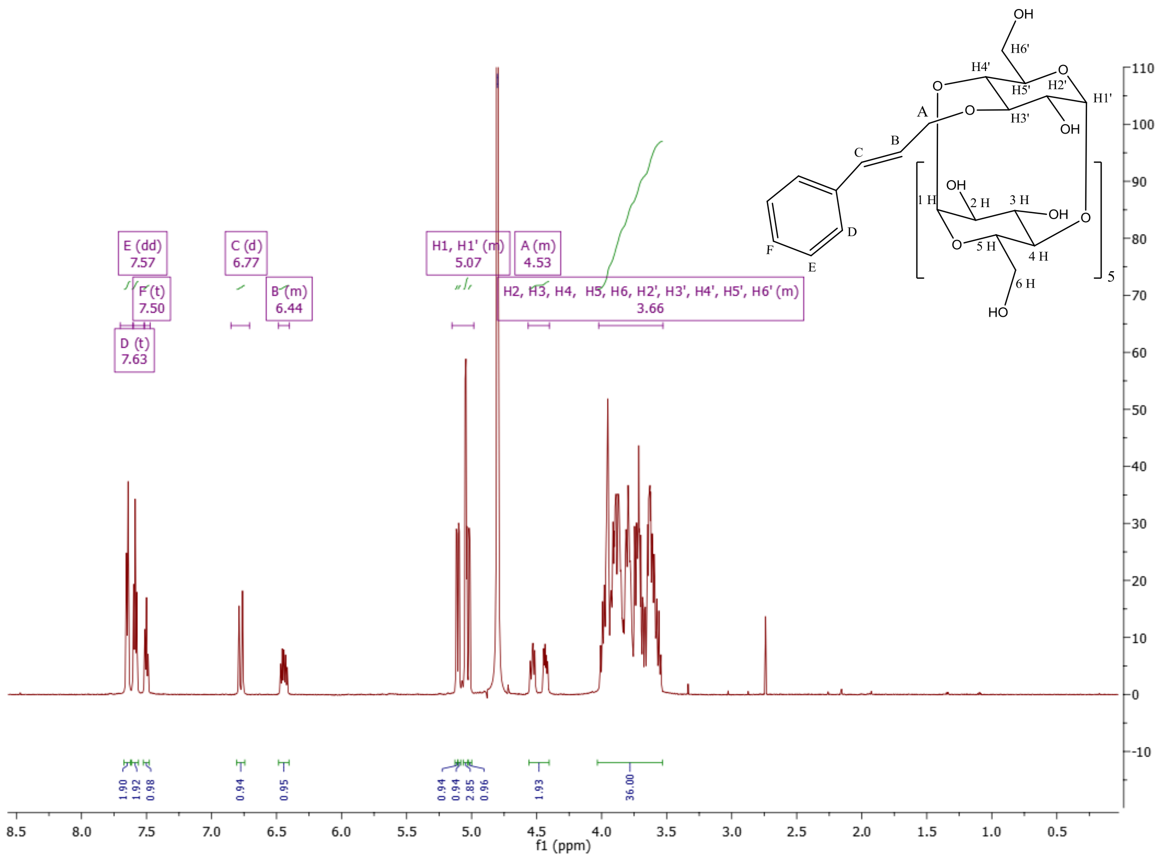

Figure SI 7: ^{1}H NMR spectrum of 3-*O*-Cin-α-CD in D_2O.

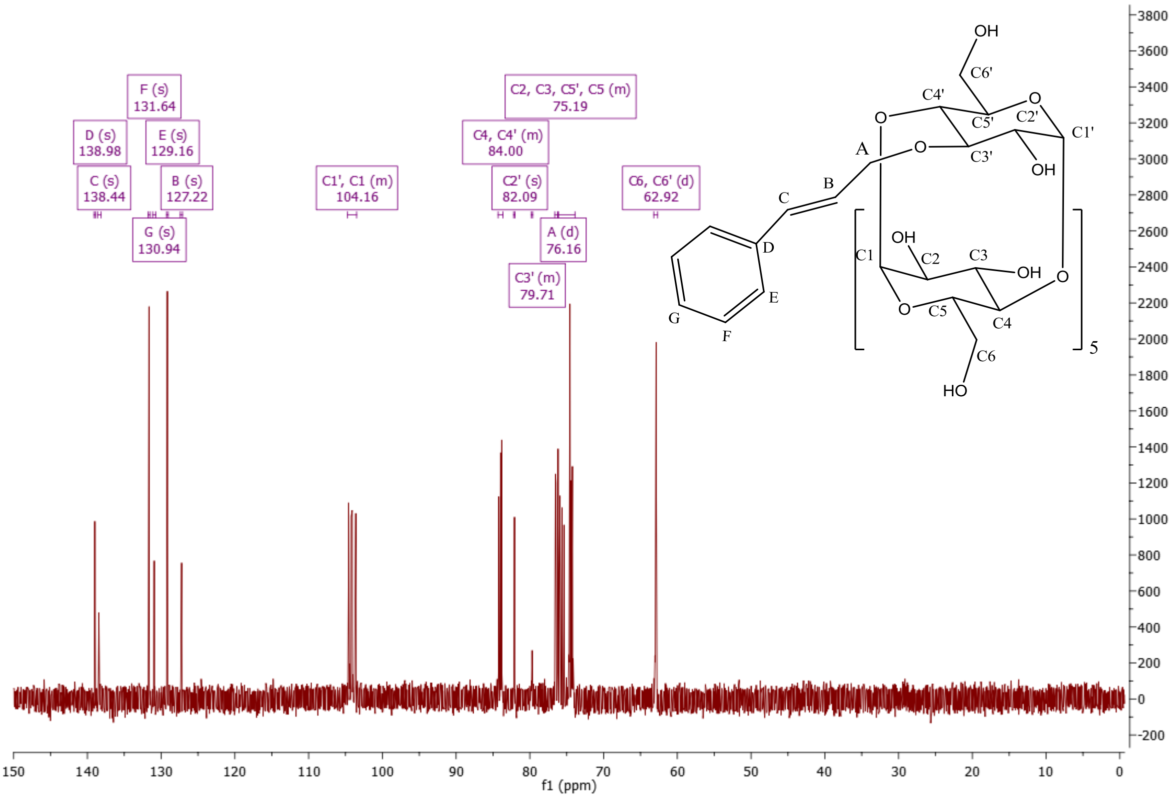

Figure SI 8: ^{13}C NMR spectrum of 3-*O*-Cin-α-CD in D_2O.

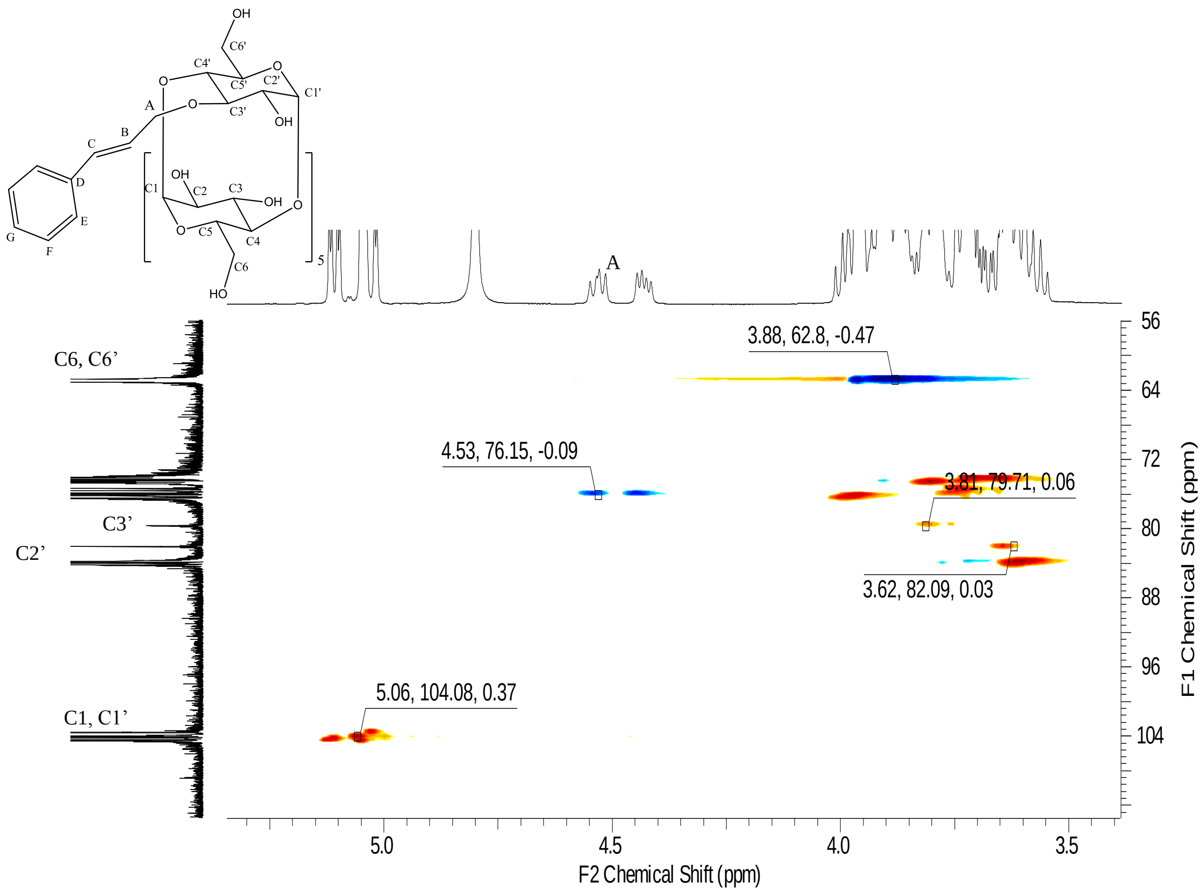

Figure SI 9: HSQC DEPT spectrum of 3-*O*-Cin-α-CD (CD region) in D_2O

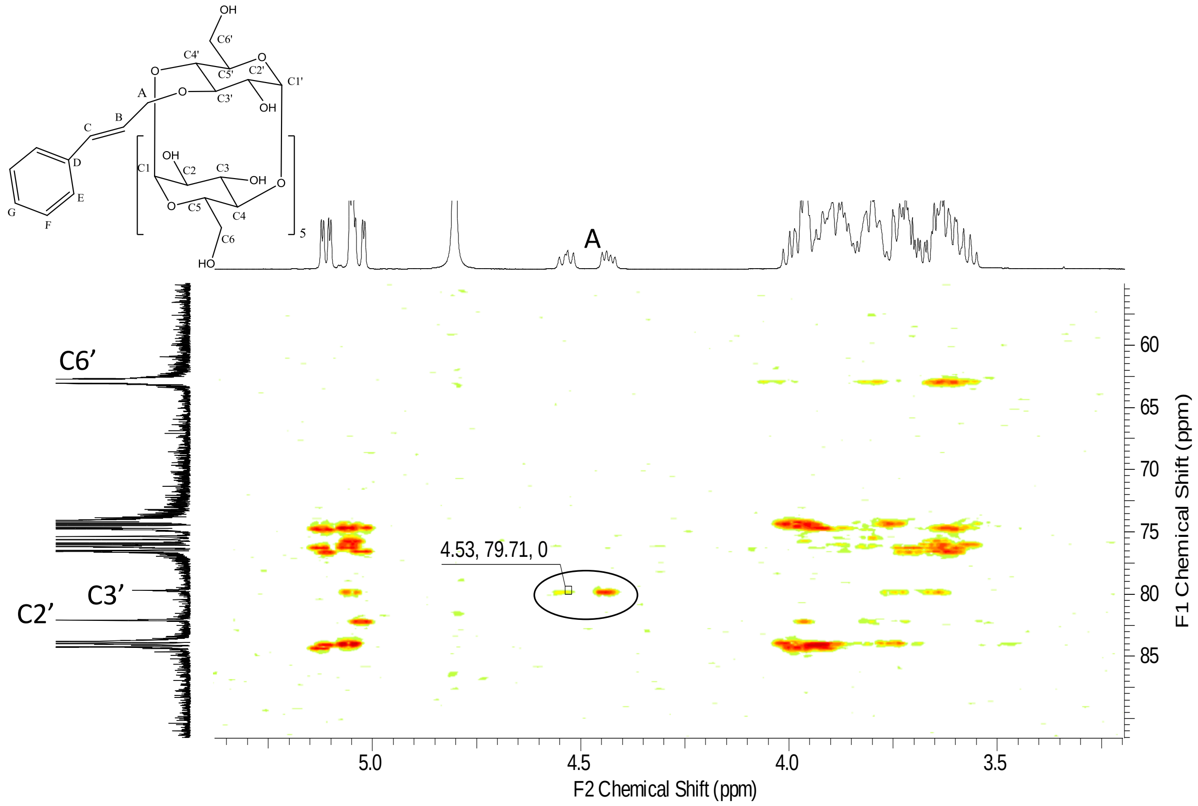

Figure SI 10: HMBC spectrum of 3-*O*-Cin-α-CD (CD region) in D_2O as a proof of the substiuent position.

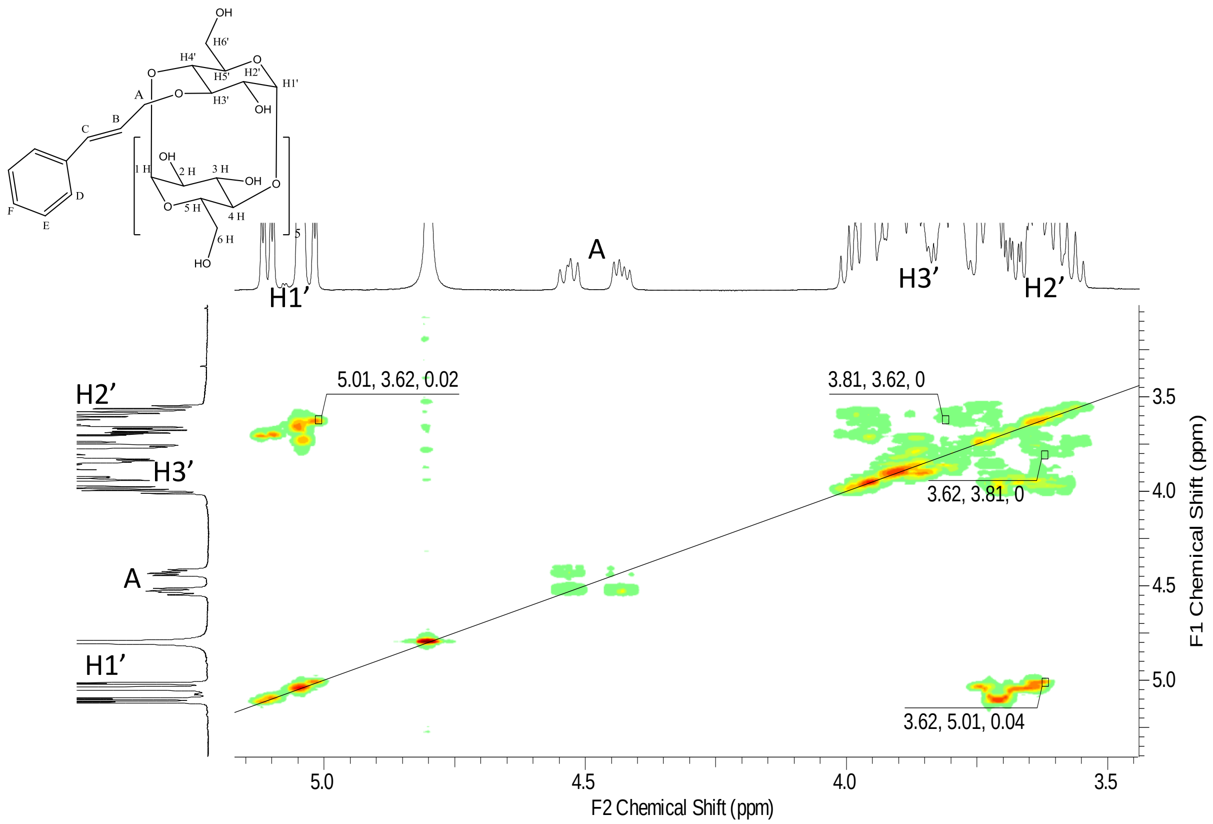

Figure SI 11: COSY spectrum of 3-*O*-Cin-α-CD (CD region) in D_2O for the identification of proton H2' and H3'.

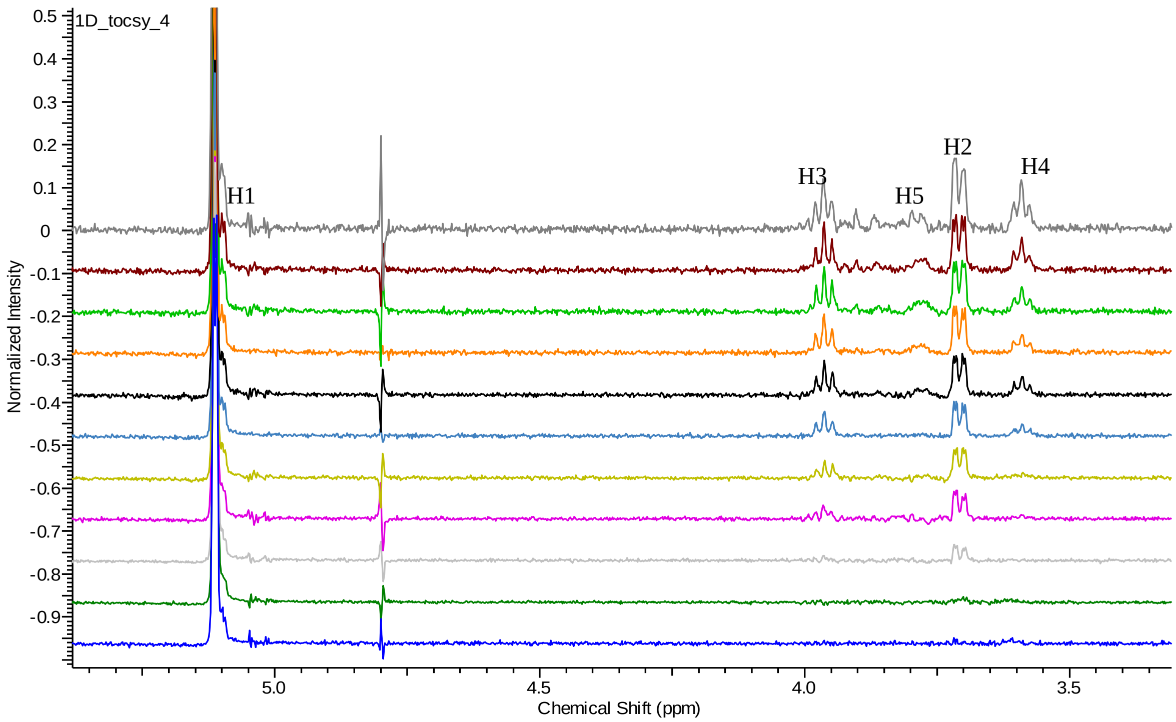

Figure SI 12: 1D TOCSY spectra of 3-*O*-Cin-α-CD (CD region) in D_2O for the identification of protons H3 and H5.

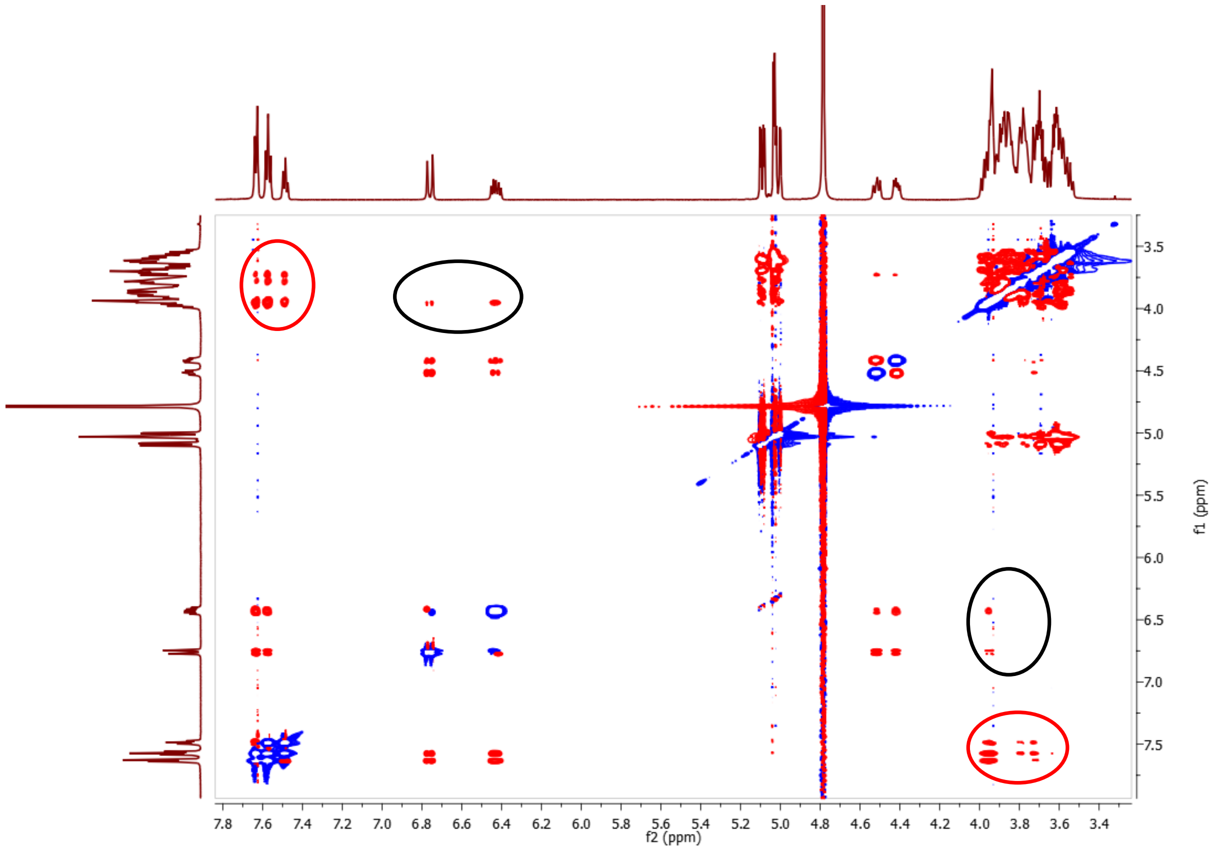

Figure SI 13: 2D ROESY spectrum of 3-*O*-Cin-α-CD in D_2O at 25 °C at 24 mM concentration.

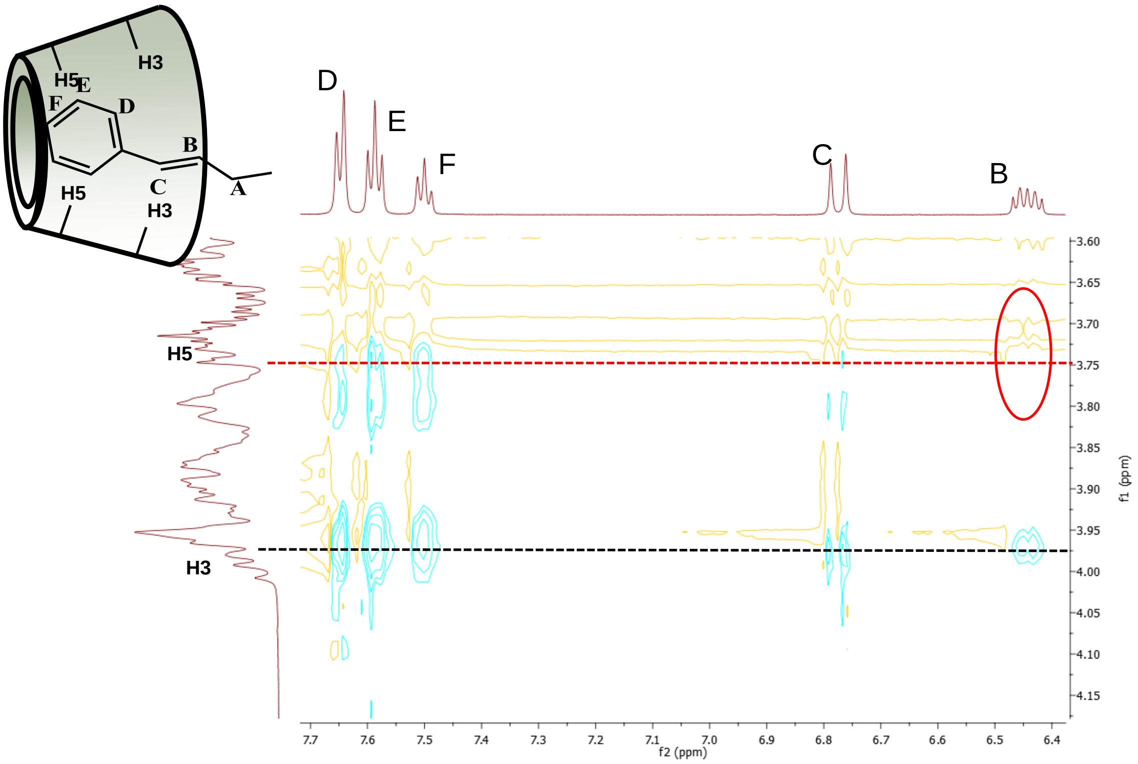

Figure SI 14: Expansion of the 2D ROESY spectrum of 3-*O*-Cin-α-CD indicating the geometric arrangement.

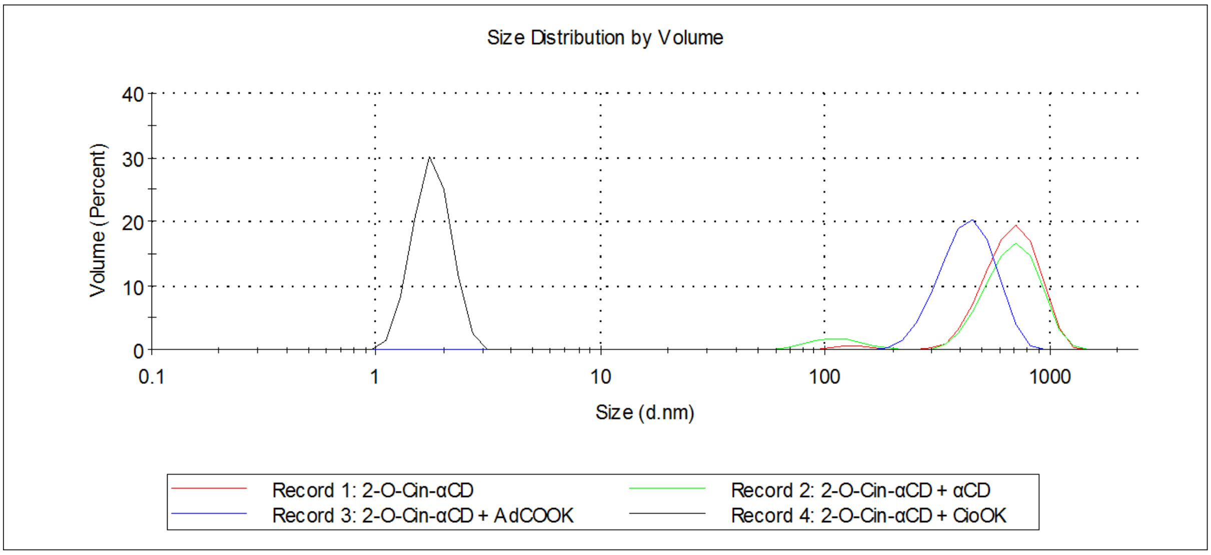

Figure SI 15: Effect of competitive host and guest molecules on the size distribution of the aggregates formed by 2-*O*-Cin-α-CD (the applied concentrations of the 2-*O*-Cin-α-CD are 10 mg/mL (9.2 mM)).

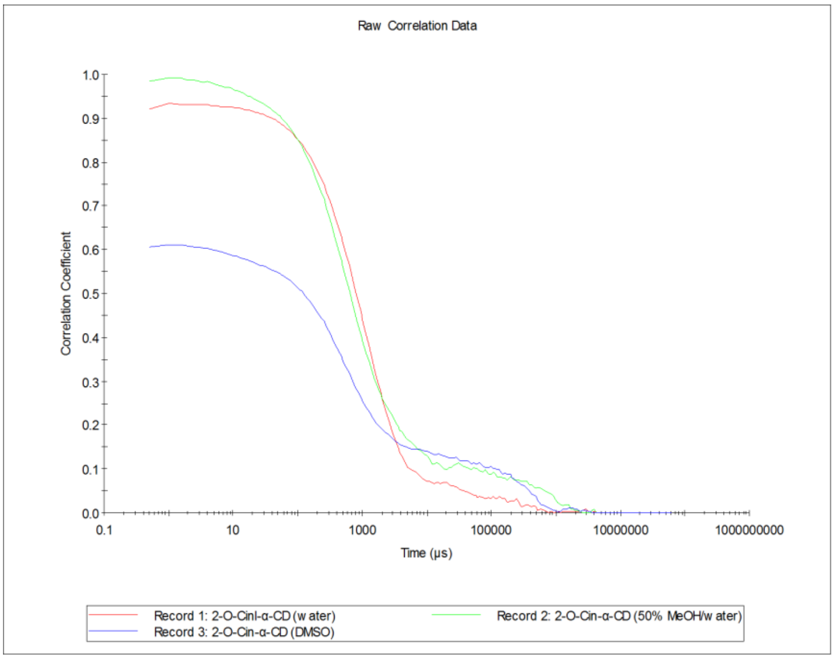

Figure SI 16: Autocorrelation functions for DLS experiment: Effect of solvent on the size distribution of aggregates formed by 2-*O*-Cin-α-CD at 25 °C.

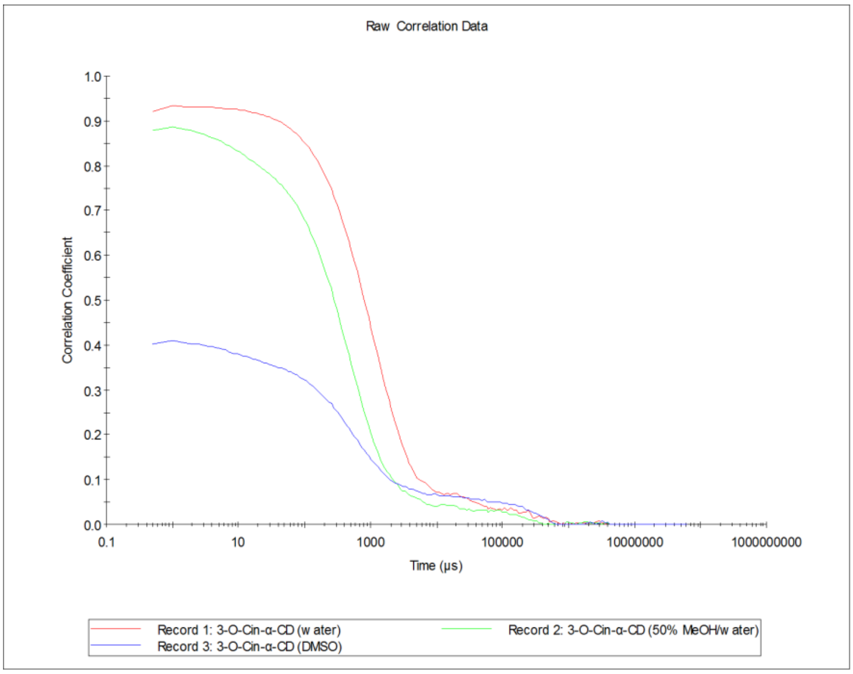

Figure SI 17: Autocorrelation functions for DLS experiment: Effect of solvent on the size distribution of aggregates formed by 3-*O*-Cin-α-CD at 25 °C.

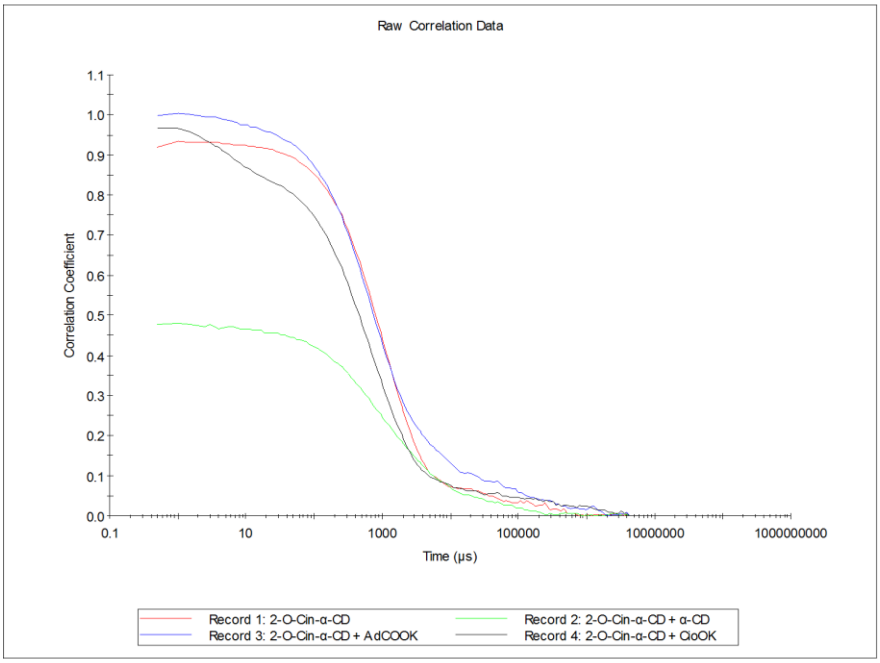

Figure SI 18: Autocorrelation functions for DLS experiment: Effect of competitive host and guest molecules on the size distribution of aggregates formed by 2-*O*-Cin-α-CD at 25 °C.

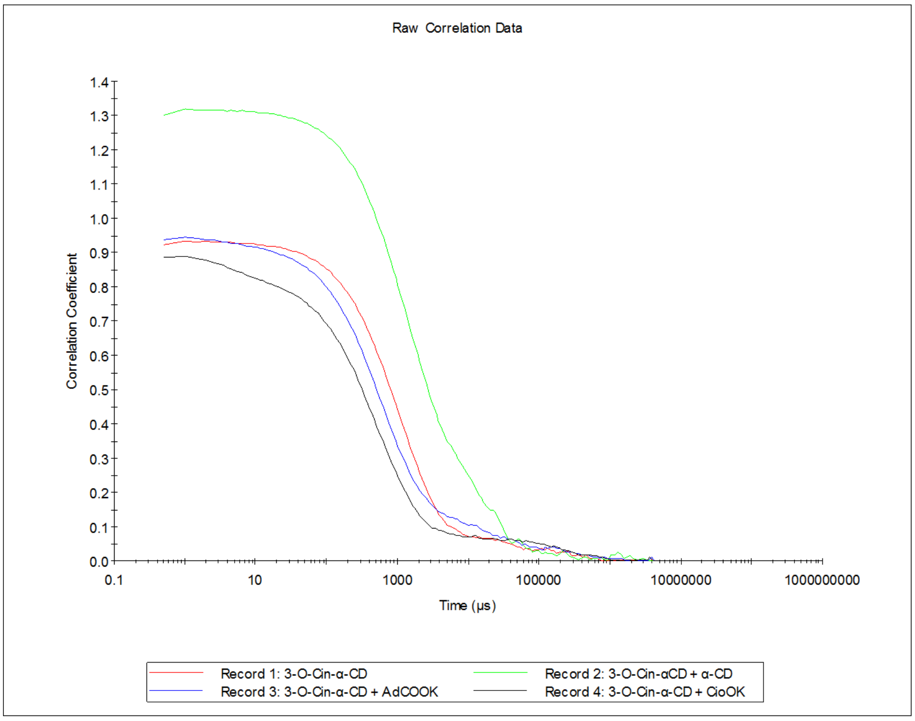

Figure SI 19: Autocorrelation functions for DLS experiment: Effect of competitive host and guest molecules on the size distribution of aggregates formed by 3-*O*-Cin-α-CD at 25 °C.

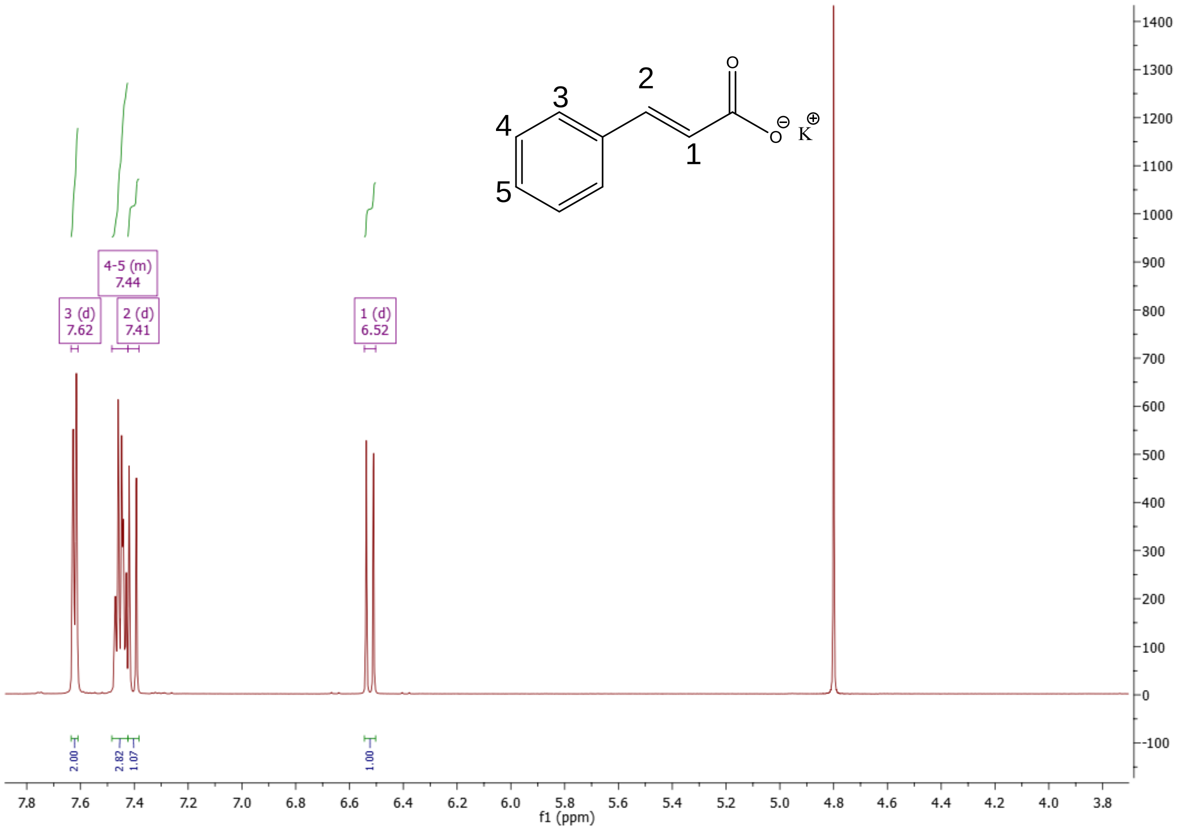

Figure SI 20: ^{1}H NMR spectrum of CioOK in D_2O at 25°C.

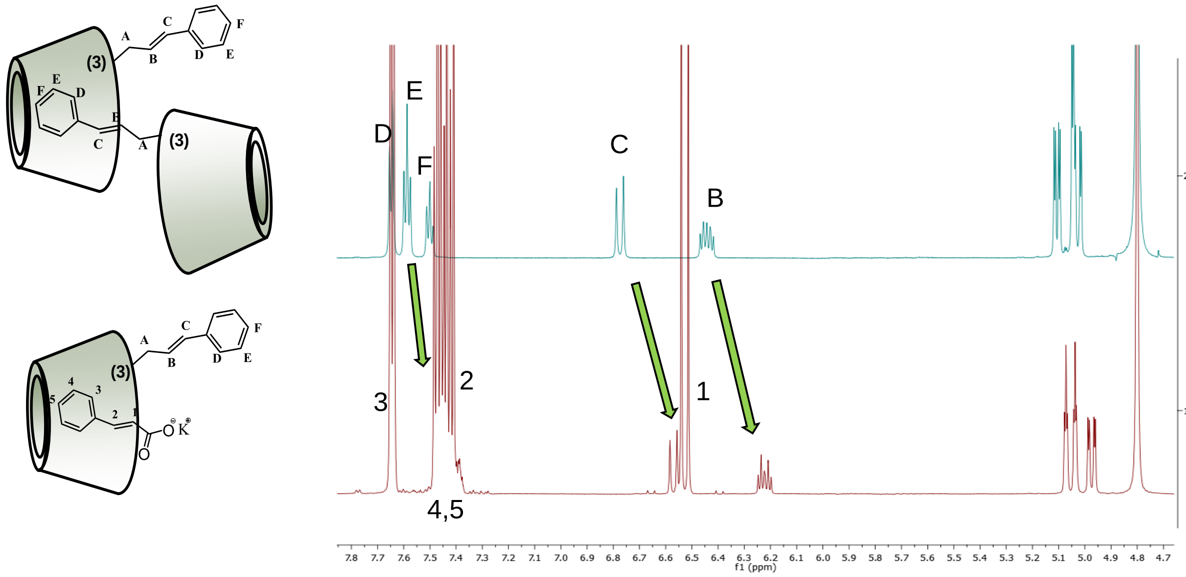

Figure SI 21: ^{1}H NMR spectrum of 3-*O*-Cin-α-CD before (up) and after (down) the addition of CioOK in 5-fold molar excess in D_2O.
